# Enantioselective *N*-heterocyclic carbene-catalyzed nucleophilic dearomatization of alkyl pyridiniums†
†Electronic supplementary information (ESI) available: Experimental conditions and compound characterization. CCDC 1555492. For ESI and crystallographic data in CIF or other electronic format see DOI: 10.1039/c7sc02648j



**DOI:** 10.1039/c7sc02648j

**Published:** 2017-08-03

**Authors:** Darrin M. Flanigan, Tomislav Rovis

**Affiliations:** a Department of Chemistry , Colorado State University , Fort Collins , CO 80523 , USA

## Abstract

NHC-catalyzed nucleophilic dearomatization of alkyl pyridiniums has been achieved to generate 1,4-dihydropyridines with high enantioselectivity. This is a rare example of catalytic, asymmetric addition of a nucleophile to the activated pyridinium that prefers C-4 functionalization leading to the 1,4-dihydropyridine with high selectivity.

## Abstract 2

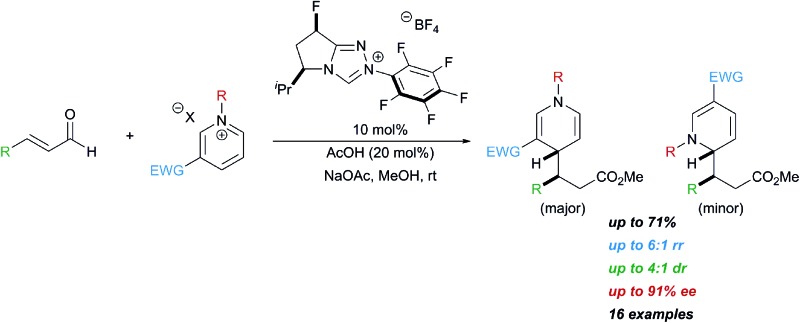
NHC-catalyzed nucleophilic dearomatization of alkyl pyridiniums has been achieved to generate 1,4-dihydropyridines with high enantioselectivity.

## Introduction

1,4-Dihydropyridines are an important class of pharmaceuticals that are used to treat an assortment of illnesses and have demonstrated antimicrobial, anticancer, antihypertensive, and anticonvulsant activity.^1^ Aside from being a privileged structure in drug discovery, 1,4-dihydropyridines are also useful intermediates en route to other substituted 6-membered heterocycles, such as pyridines or piperidines.^2^ Despite the importance of the 1,4-dihydropyridine scaffold, direct and mild methods for their synthesis remains a significant challenge.

**Scheme 1:**
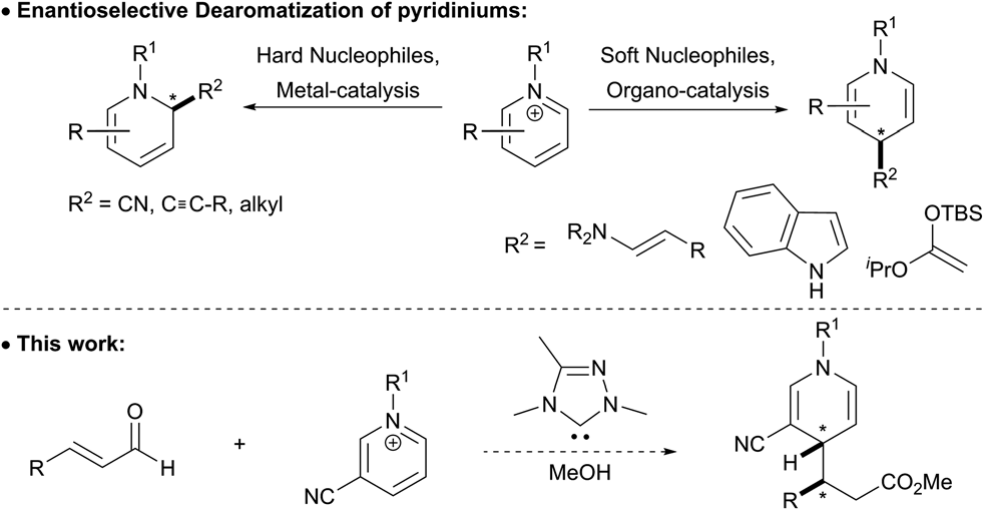
Asymmetric synthesis of dihydropyridines.

Historically, the synthesis of 1,4-dihydropyridines has relied on condensation reactions, *e.g.* the Hantzsch reaction.^3^ The nucleophilic dearomatization of activated pyridines has emerged as a powerful tool to synthesize a variety of 6-membered nitrogen containing heterocycles.^4^ However, many of these methods require chiral auxiliaries on the pyridine derivative to direct the approach of the incoming nucleophile, which limits the broad application of this methodology.^5^ More recently, much effort has been focused on catalytic enantioselective methods that can directly access dihydropyridines stereoselectively.^6^ Transition metal-catalyzed reactions have been applied to the asymmetric synthesis of dihydropyridines and typically generate 1,2-dihydropyridines (Scheme 1). Anion-binding catalysis has been demonstrated to be an effective catalytic asymmetric strategy for nucleophilic dearomatization of *N*-Troc pyridiniums with silyl ketene acetals to give primarily 1,2-dihydropyridines.^7^ This method was extended to coupling indoles with pyridiniums, which gives the 1,4-dihydropyridine with high regioselectivity.^8^ Secondary amine catalysis has also been employed to synthesize 1,4-dihydropyridines from *N*-alkyl pyridiniums bearing a nitro group at the 3-position.^9^ To achieve high diastereo- and enantioselectivity in these reactions, a two-step protocol was developed where the product aldehyde was immediately trapped *in situ* with Wittig reagents to give the γ-substituted α,β-unsaturated ester in good yield, with high enantio- and diastereoselectivity.

*N*-Hetereocyclic carbenes (NHCs) are versatile organocatalysts that have been used in a variety of catalytic asymmetric reactions.^10^ Our group^11^ and others^12^ have successfully employed NHC-catalyzed functionalization of enals resulting in carbon–carbon bond formation at the beta position of the aldehyde to give β-functionalized esters. We wondered if the homoenolate intermediate was nucleophilic enough to intercept pyridinium derivatives, and if this nucleophile would selectively add to the 4-position of the pyridinium.

We began optimizing the reaction by screening several chiral catalysts (Table 1). The triazolium scaffolds tested in the reaction demonstrate high tunability of the enantioselectivity, depending on the catalyst structure. However, the regio- and diastereoselectivity was less sensitive to the choice of catalyst. Initially, the product was generated in low yields (entries 1–4), but yields were improved when oxygen was rigorously excluded from the reaction (entries 5 & 6). At this point, we wondered if the reaction could be carried out with lower catalyst loadings. Attempting the reaction with 10 mol% catalyst loading gives the product in 27% yield. To increase the yield of the process, we screened a variety of additives in the reaction and acetic acid (20 mol%) was found to be beneficial to the reactivity, giving the product in 61% yield, without affecting the selectivity of the process.

**Table 1:** Optimization of the Reaction^*a*^

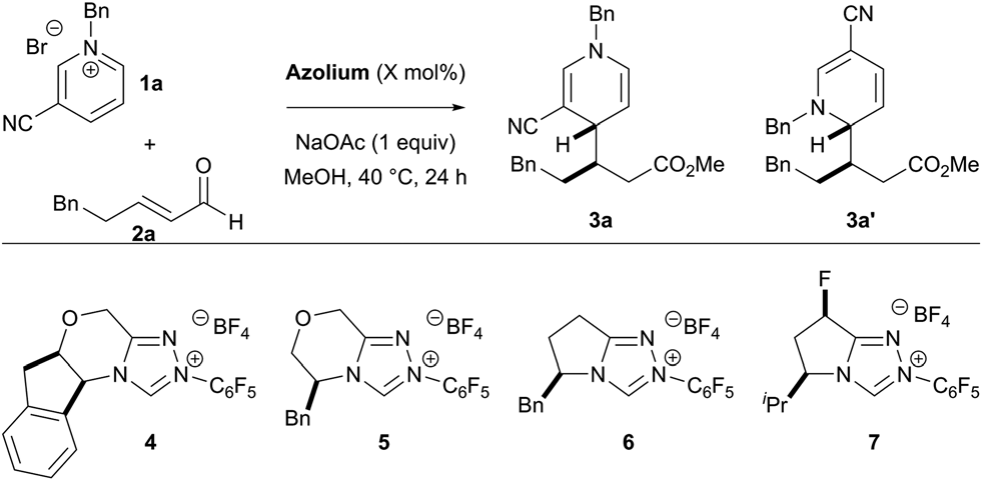						
Entry	Catalyst	X	Yield^*b*^ [%]	**3a** : **3a′** ^*c*^	dr^*d*^	ee^*e*^ [%]
1	**4**	20	29	6 : 1	2 : 1	53
2	**5**	20	30	3 : 1	3 : 1	87
3	**6**	20	14	5 : 1	3 : 1	88
4	**7**	20	40	6 : 1	3 : 1	87
5^*f*^	**7**	20	87	6 : 1	3 : 1	87
6^*f*^	**7**	10	27	6 : 1	3 : 1	87
**7** ^*f*^ ^,^ ^*g*^	**7**	**10**	**61**	**6** **:** **1**	**3** **:** **1**	**87**

^*a*^Reaction conducted with 1.5 equiv. of **1a** and 1.0 equiv. of **2a**.

^*b*^Combined yield of **3a** and **3a′**; determined by ^1^H NMR using 1,3,5-trimethoxybenzene as an internal standard.

^*c*^Regioisomeric ratio determined by ^1^H NMR of the unpurified reaction mixture.

^*d*^Diastereomeric ratio of **3a**; determined by ^1^H NMR of the unpurified reaction mixture.

^*e*^Enantiomeric excess determined by HPLC using a chiral stationary phase.

^*f*^Reaction set up in glovebox.

^*g*^Reaction conducted in presence of 20 mol% acetic acid.

**Scheme 2:**
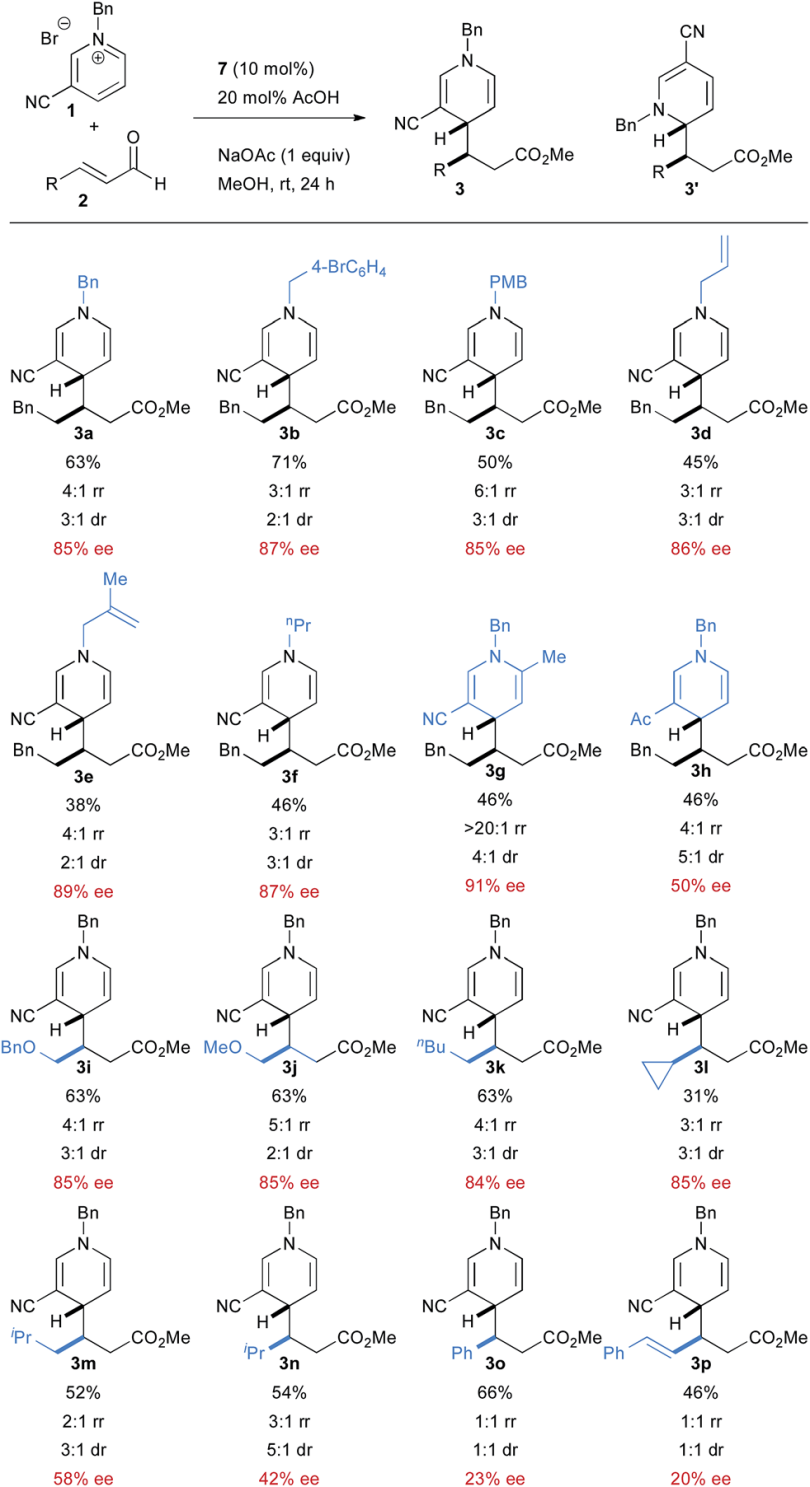
Reaction scope.

With optimized conditions in hand, we then explored the scope of the reaction. A broad scope of *N*-alkyl groups are tolerated on the pyridinium (Scheme 2). *N*-acyl pyridiniums, on the other hand, are not competent substrates. An electron withdrawing group at the 3 position of the pyridinium is required to achieve good reactivity with the cyano group providing the product with the highest ee. Acetyl pyridinium **1h** is tolerated, but leads to much lower enantioselectivity, although the diastereoselectivity and regioselectivity are improved compared to the cyano group. 2-Methyl-5-cyano pyridinium is tolerated in the reaction, providing very high regioselectivity. A variety of aliphatic enals are tolerated in the reaction although enantioselectivity suffers with increasing size of the alkyl group. Straight-chain aliphatic enals proceed with the highest enantioselectivity. Interestingly, a cyclopropyl enal is also well tolerated.

Cinnamaldehyde is a competent coupling partner and proceeds with good yield, but with much lower levels of selectivity. The same is true with conjugated enals, which provide the product with good yield, but with no regio- or diastereoselectivity (and nearly racemic product). The reasons for this profound decrease in selectivity are not clear.

**Scheme 3:**
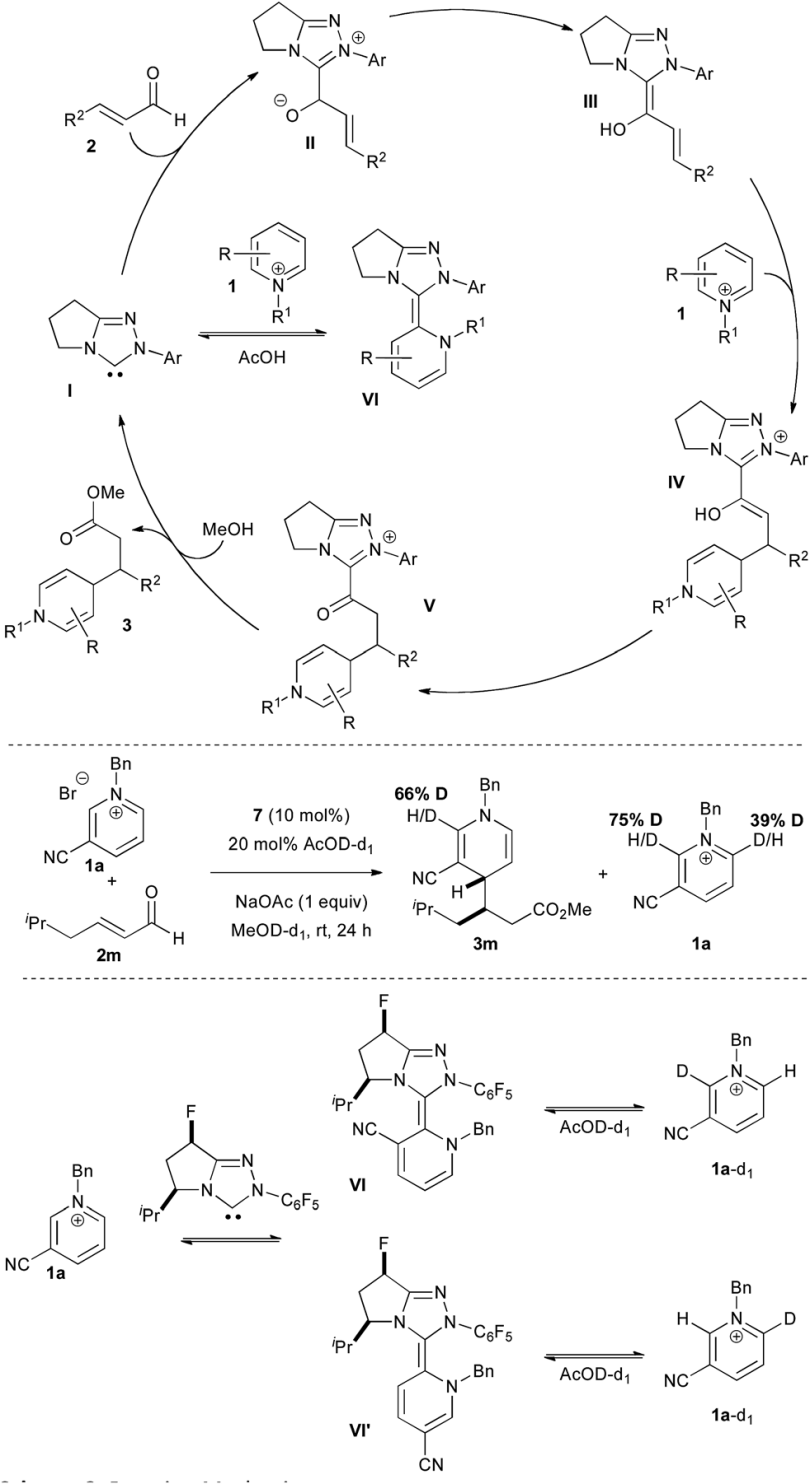
Reaction mechanism.

The mechanism of the reaction is illustrated in Scheme 3. The carbene catalyst **I** adds to the enal to ultimately generate the Breslow intermediate **III**. Addition to pyridinium **1** forms the enol azolium **IV** which tautomerizes to keto azolium **V**. Methanolysis leads to catalyst turnover and product formation.

The extreme sensitivity of this reaction to the presence of oxygen and the need for acetic acid additive for improved yields puzzled us. Most NHC-catalyzed reactions are air and moisture tolerant. Indeed, we have shown that aqueous conditions may be used with α-fluoroenals to deliver the corresponding acids directly.^13^ We speculated that the NHC catalyst may be adding to the pyridinium salt directly in a consumptive off-cycle path (**I** to **VI**, Scheme 3). The presence of acetic acid provides the buffer and acid needed to regenerate the carbene catalyst. We have previously noted such behaviour in the addition of carbene to iminium electrophiles.^14,15^

Support for this mechanism was gained from an isotope labelling experiment. Subjection of the reaction to AcOD in MeOD resulted in deuterium uptake in the dihydropyridine product as well as deuteration in the unreacted pyridinium starting material (Scheme 3). The latter indicates that both the 2- and 6-positions of the pyridinium are deuterated, likely resulting from carbene addition to form the aza-Breslow intermediates **VI** and **VI'**.

**Scheme 4:**
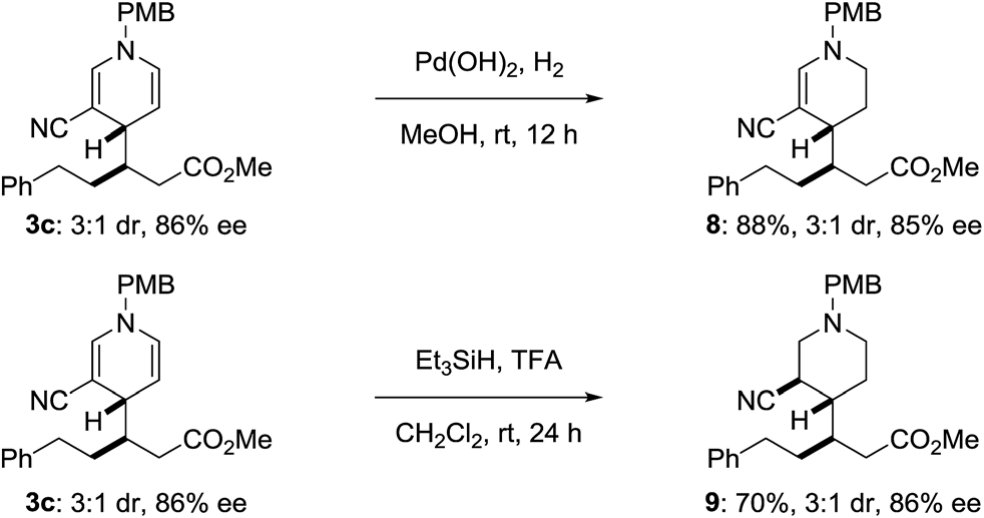
Product derivatization.

The product dihydropyridines are useful target structures but may also be used toward the synthesis of other heterocyclic scaffolds. Treating dihydropyridine **3c** with palladium hydroxide and H_2_ in methanol generates the tetrahydropyridine **8** (Scheme 4), selectively reducing the more electron rich double bond. The dihydropyridine could also be reduced by treating **3c** with excess triethylsilane and trifluoroacetic acid, without eroding the pre-existing stereocenters, delivering piperidine **9** (ref. 16) with three contiguous stereocenters.

## Conclusion

In conclusion, we have developed an enantio- and diastereoselective addition of enals to pyridiniums using NHC-catalysis. The 1,4-dihydropyridine products are generated with good regiocontrol over the 1,2-addition product and with high enantioselectivity. Key to the success of this reaction was the addition of catalytic amounts of acetic acid to improve the yield of the reaction, preventing an off-cycle catalyst-pyridinium adduct trap.
