# Supplementary material for: Enantioselective N-heterocyclic carbene-catalyzed nucleophilic dearomatization of alkyl pyridiniums
Source: Chem Sci. 2017 Aug 3;8(9):6566–9. doi: 10.1039/c7sc02648j (PMC5627355; doi:10.1039/c7sc02648j)
Supplement: Supplementary file 1 [file SC-008-C7SC02648J-s001.pdf]

Electronic Supplementary Material (ESI) for Chemical Science.
This journal is © The Royal Society of Chemistry 2017

Enantioselective N-Heterocyclic Carbene-Catalyzed Nucleophilic Dearomatization of Alkyl Pyridiniums

Supporting Information

Darrin M. Flanigan[†,‡], Tomislav Rovis[†,‡,*]

[†]*Department of Chemistry, Colorado State University, Fort Collins, Colorado 80523, United States*

[‡]*Department of Chemistry, Columbia University, New York, New York 10027, United States*

Materials and Methods

All reactions were carried out with magnetic stirring under an atmosphere of argon in oven-dried glassware. Methanol was purchased from Sigma-Aldrich and stored in an anhydrous atmosphere. Sodium acetate was purchased from Aldrich and stored under anhydrous atmosphere.
^{1}H NMR spectra were recorded on Varian 400 MHz spectrometer at ambient temperature or a Bruker Avance III 500 (500 MHz). Data is reported as follows: chemical shift in parts per million (δ, ppm) from $CDCl_3$ (7.26 ppm) or acetone-D6 (2.03 ppm), multiplicity (s = singlet, bs = broad singlet, d = doublet, t = triplet, q = quartet, and m = multiplet), coupling constants (Hz). ^{13}CNMR were recorded on Varian 400 MHz (at 100 MHz) spectrometer or a Bruker Avance III 500 (125 MHz) at ambient temperature. Chemical shifts are reported in ppm from $CDCl_3$ (77.36 ppm) or acetoneD-6 (205.87, 30.6 ppm). Mass spectra were recorded on an Agilent 6130 Quadrupole LC/MS. HPLC spectra were obtained on an Agilent 1100 series system. Optical rotations were obtained on an Autopol - III automatic polarimeter or a Jasco DIP - 1000 digital polarimeter. Infrared spectra were recorded on a Perkin - Elmer Spectrum Two (Diamond ATR) IR or a Nicolet iS-50 FT-IR spectrometer. Thin layer chromatography was performed on SiliCycle® 250µm 60A plates. Visualization was accomplished with UV light or $KMnO_4$ stain followed by heating.

General Procedure for the synthesis of dihydropyridine derivatives
The 1,4-dihydropyridines were synthesized by combining 0.2 mmol of enal, 0.30 mmol pyridinium salt, 0.02 mmol NHC (10 mol%) and 0.2 mmol NaOAc in a vial equipped with a stir bar and Teflon cap. This mixture was then placed in an inert atmosphere (glove box) and diluted with 2 mL of methanol (0.1 M) along with 0.02 mmol acetic acid (20 mol%). The vial was then sealed and stirred at room temperature for 24 h. After this time, the solvent was evaporated and the crude reaction mixture was purified by silica chromatography (dry loading on celite) to afford the title compounds.

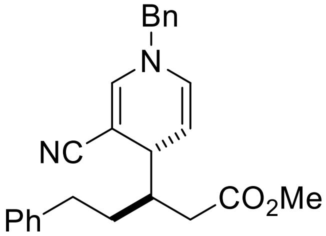

methyl 3-(1-benzyl-3-cyano-1,4-dihydropyridin-4-yl)-5-phenylpentanoate (**3a**): Pale yellow oil. 61% yield, 5:1 rr, 3:1 dr, 88% ee. R_f = 0.3 (4:1 Hexanes:EtOAc); $[\alpha]_D^{21}$ = -32.8 (c = 0.136 g/mL); **HPLC analysis:** Chiralpak IA column, 80:20 hexanes/iso-propanol, 0.5 mL/min. Major: 18.1 min, minor: 16.8. **^{1}HNMR**: (500 MHz, Chloroform-d) δ 7.31 (dd, J = 4.7, 2.5 Hz, 5H), 7.23 (d, J = 6.0 Hz, 3H), 7.17 – 7.14 (m, 2H), 6.74 (d, J = 1.5 Hz, 1H), 5.89 (d, J = 8.1 Hz, 1H), 4.63 (ddd, J = 7.2, 4.4, 2.7 Hz, 1H), 4.29 (s, 2H), 3.69 (s, 3H), 3.48 (t, J = 3.7 Hz, 1H), 2.69 – 2.65 (m, 2H), 2.41 (dd, J = 7.1, 4.1 Hz, 2H), 2.13 – 1.98 (m, 2H), 1.62 (qd, J = 5.5, 2.8 Hz, 2H); **^{13}CNMR:** (101 MHz, Chloroform-d) δ 173.51, 144.24, 142.21, 136.07, 129.55, 129.02, 128.41, 127.12, 121.23, 103.51, 57.55, 51.68, 51.62, 41.73, 36.37, 35.66, 33.87, 33.04; **IR** (ATR, neat) 3026, 2923, 2856, 2191, 1730, 1672, 1590, 1412, 1181, 735, 701 cm^{-1} **LRMS** (ESI + APCI) *m/z* [M+H] calcd 387.2, found 387.2

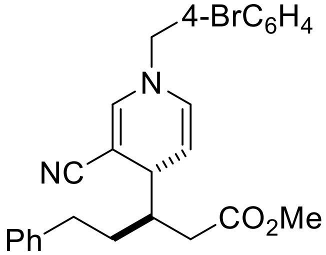

methyl 3-(1-benzyl-3-cyano-1,4-dihydropyridin-4-yl)-5-methylhexanoate (**3b**): Pale yellow oil. 71% yield, 3:1 rr, 2:1 dr, 87% ee. R_f = 0.35 (4:1 Hexanes:EtOAc); $[\alpha]_D^{21}$ = -12.2 (c = 0.01 g/mL); **HPLC analysis:** Chiralpak IA column, 90:10 hexanes/iso-propanol, 1.0 mL/min. Major: 11.2 min, minor: 10.8 min. **^{1}HNMR:** (400 MHz, Chloroform-d) δ 7.33 (dd, J = 25.5, 7.9 Hz, 3H), 7.25 – 7.04 (m, 3H), 6.99 (d, J = 8.4 Hz, 2H), 6.69 (d, J = 1.6 Hz, 1H), 5.91 – 5.76 (m, 1H), 4.61 (dd, J = 8.1, 4.4 Hz, 1H), 4.21 (s, 2H), 3.67 (s, 3H), 3.52 – 3.40 (m, 1H), 2.80 – 2.56 (m, 2H), 2.42 – 2.27 (m, 2H), 2.21 – 1.92 (m, 2H), 1.76 – 1.47 (m, 2H); **^{13}CNMR:** (101 MHz, Chloroform-d) δ 173.33, 143.92, 142.15, 129.30, 128.36, 125.91, 122.19, 120.89, 103.67, 103.01, 81.70, 81.22, 56.91, 51.67, 41.57, 41.21, 36.40, 35.57, 33.86, 33.16; **IR** (ATR, neat) 3060, 3025, 2923, 2857, 2191, 1729, 1672, 1591, 1435, 1404, 1178, 1010 cm^{-1} **LRMS** (ESI + APCI) *m/z* [M+H] calcd 465.1, found 465.1

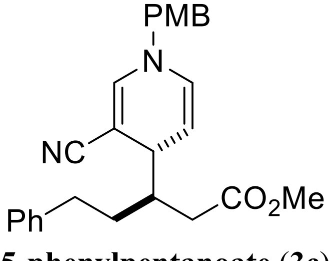

methyl 3-(3-cyano-1-(4-methoxybenzyl)-1,4-dihydropyridin-4-yl)-5-phenylpentanoate (**3c**): Pale yellow oil. 50% yield, 6:1 rr, 3:1 dr, 85% ee. R_f = 0.3 (4:1 Hexanes:EtOAc); $[\alpha]_D^{21}$ = -22.2 (c = 0.01 g/mL); **HPLC analysis:** Chiralpak IA column, 90:10 hexanes/iso-propanol, 1.0 mL/min. Major: 12.0 min, minor: 10.8 min. **^{1}HNMR**: (400 MHz, Chloroform-d) δ 7.33 – 7.27 (m, 3H), 7.23 – 7.15 (m, 5H), 7.09 (d, J = 8.8 Hz, 2H), 7.05 (d, J = 8.8 Hz, 2H), 6.86 (d, J = 8.7 Hz, 2H), 6.79 (d, J = 8.7 Hz, 2H), 6.70 (d, J = 1.6 Hz, 1H), 5.86

(ddd, J = 8.2, 1.7, 0.9 Hz, 1H), 4.59 (dd, J = 8.1, 4.4 Hz, 1H), 4.19 (s, 2H), 3.79 (s, 3H), 3.66 (s, 3H), 3.46 – 3.41 (m, 1H), 2.80 – 2.59 (m, 4H), 2.34 – 2.22 (m, 1H), 2.15 – 1.94 (m, 3H), 1.74 – 1.50 (m, 3H); **^{13}CNMR:** (101 MHz; CDCl3): δ 173.5, 159.5, 144.1, 142.2, 129.4, 128.5, 128.4, 128.4, 127.9, 121.3, 114.4, 103.4, 80.4, 57.1, 55.3, 51.6, 41.8, 41.3, 36.4, 35.6, 33.9, 33.0; **IR** (ATR, neat) 3061, 3026, 2925, 2856, 2191, 1731, 1672, 1588, 1513, 1412, 1248, 1175 cm^{-1} **LRMS** (ESI + APCI) *m/z* [M+H] calcd 417.2, found 417.2

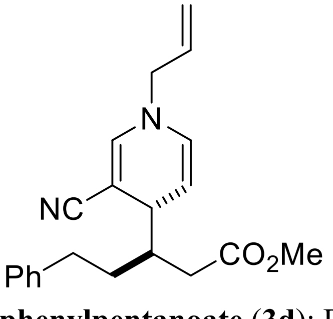

methyl 3-(1-allyl-3-cyano-1,4-dihydropyridin-4-yl)-5-phenylpentanoate (**3d**): Pale yellow oil. 45% yield, 3:1 rr, 3:1 dr, 86% ee. R_f = 0.2 (4:1 Hexanes:EtOAc); $[\alpha]_D^{21}$ = -19.3 (c = 0.01 g/mL); **HPLC analysis:** Chiralpak IA column, 90:10 hexanes/iso-propanol, 1.0 mL/min. Major: 16.0 min, minor: 14.5 min. **^{1}HNMR:** (400 MHz, Chloroform-d) δ 7.30 – 7.28 (m, 3H), 7.21 – 7.16 (m, 2H), 6.64 (d, J = 4 Hz, 1H), 5.71 – 5.68 (m, 1H), 5.84 – 5.82 (m, 1H), 5.25 – 5.17 (m, 2H), 4.58 (dd, J = 4, 8 Hz, 1H), 3.66 (s, 3H), 3.47 – 3.43 (m, 1H), 2.75 – 2.62 (m, 3H), 2.45 – 2.25 (m, 2H), 2.05 – 1.96 (m, 2H), 1.68 – 1.56 (m, 2H); **^{13}CNMR:** (101 MHz; CDCl3): δ 173.50, 148.26, 143.98, 142.14, 132.68, 129.29, 128.36, 125.78, 121.27, 118.66, 109.60, 103.41, 102.07, 80.26, 56.13, 21.61, 41.91, 41.20, 36.26, 35.69, 33.79, 33.63, 32.97, 32.55; **IR** (ATR, neat) 3061, 3025, 2925, 2860, 2192, 1673, 1591, 1412, 1218, 1189 cm^{-1} **LRMS** (ESI + APCI) *m/z* [M+H] calcd 337.2, found 337.2

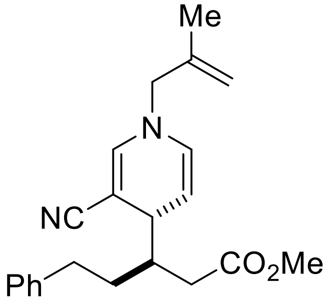

methyl 3-(3-cyano-1-(3-methylbut-3-en-1-yl)-1,4-dihydropyridin-4-yl)-5-phenylpentanoate (**3e**): Pale yellow oil. 38% yield, 4:1 rr, 2:1 dr, 89% ee. R_f = 0.3 (4:1 Hexanes:EtOAc); $[\alpha]_D^{21}$ = 23.1 (c = 0.009 g/mL); **HPLC analysis:** Chiralpak OC column, 90:10 hexanes/iso-propanol, 1.0 mL/min. Major: 40.0 min, minor: 37.8 min. **1HNMR:** (400 MHz, Chloroform-d) δ 7.25 (d, J = 3.1 Hz, 1H), 7.23 – 7.12 (m, 4H), 6.59 (dd, J = 10.7, 1.6 Hz, 1H), 5.90 – 5.80 (m, 1H), 4.86 – 4.63 (m, 3H), 4.57 (dd, J = 8.1, 4.4 Hz, 1H), 3.66 (s, 3H), 3.48 – 3.40 (m, 1H), 3.20 (td, J = 7.1, 4.4 Hz, 2H), 2.66 – 2.57 (m, 2H), 2.37 (dd, J = 17.3, 7.0 Hz, 1H), 2.23 – 2.15 (m, 3H), 2.06 – 1.94 (m, 2H), 1.70 (d, J = 12.7 Hz, 4H); **13CNMR:** (101 MHz, Chloroform-d) δ 173.55, 144.01, 142.19, 140.81, 129.14, 128.37, 125.77, 123.61, 121.49, 113.57, 109.19, 103.32, 79.47, 52.42, 51.60, 42.01, 41.30, 37.92, 36.15, 35.65, 33.82, 33.62, 32.97, 32.46, 22.18; **IR** (ATR, neat) 3061, 3026, 2925, 2857, 1731, 1672, 1625, 1588, 1414, 1170 cm^{-1}; **LRMS** (ESI + APCI) *m/z* [M+H] calcd 365.2, found 365.2

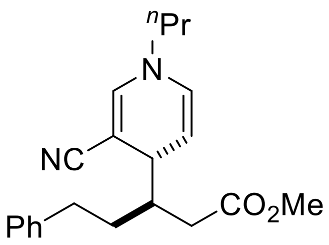

methyl 3-(3-cyano-1-propyl-1,4-dihydropyridin-4-yl)-5-phenylpentanoate (**3f**): Pale yellow oil. 46% yield, 3:1 rr, 3:1 dr, 88% ee. R_f = 0.3 (4:1 Hexanes:EtOAc); $[\alpha]_D^{21}$ = -34.0 (c = 0.01 g/mL); **HPLC analysis:** Chiralpak IA column, 90:10 hexanes/iso-propanol, 1.0 mL/min. Major: 13.0 min, minor: 11.7 min. ^{1}**HNMR**: (400 MHz, Chloroform-d) δ 7.33 – 7.27 (m, 1H), 7.23 – 7.11 (m, 3H), 6.63 (d, J = 1.6 Hz, 1H), 5.90 – 5.80 (m, 1H), 4.60 – 4.53 (m, 1H), 3.66 (s, 3H), 3.48 – 3.40 (m, 1H), 3.08 – 2.98 (m, 2H), 2.67 – 2.56 (m, 2H), 2.48 – 2.30 (m, 2H), 2.08 – 1.92 (m, 2H), 1.57 – 1.49 (m, 2H), 0.86 (t, J = 7.4 Hz, 3H); ^{13}**CNMR:** (101 MHz; CDCl3): δ 173.56, 144.17, 142.17, 129.31, 128.34, 123.61, 121.59, 101.85, 79.13, 55.86, 51.60, 42.02, 41.29, 36.19, 35.65, 33.78, 32.89, 23.14, 10.79; **IR** (ATR, neat) 3061, 3026, 2930, 2876, 2190, 1731, 1671, 1587, 1414, 1133 cm^{-1} **LRMS** (ESI + APCI) *m/z* [M+H] calcd 339.2, found 339.2.

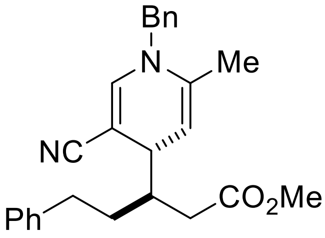

methyl 3-(1-benzyl-5-cyano-2-methyl-1,4-dihydropyridin-4-yl)-5-methylhexanoate (**3g**): Pale yellow oil. 46% yield, >20:1 rr, 4:1 dr, 91% ee. R_f = 0.3 (4:1 Hexanes:EtOAc); $[\alpha]_D^{21}$ = -11.6 (c = 0.01 g/mL); **HPLC analysis:** Chiralpak IA column, 90:10 hexanes/iso-propanol, 1.0 mL/min. Major: 15.0 min, minor: 13.6 min. ^{1}**HNMR**: (400 MHz, Chloroform-d) δ 7.36 – 7.27 (m, 3H), 7.26 – 7.14 (m, 6H), 7.11 (dd, J = 6.5, 3.1 Hz, 1H), 6.73 (s, 1H), 4.53 – 4.32 (m, 3H), 3.67 (s, 3H), 3.51 – 3.42 (m, 1H), 2.78 – 2.58 (m, 3H), 2.40 (dd, J = 7.0, 2.5 Hz, 1H), 2.16 – 1.95 (m, 2H), 1.75 (s, 3H), 1.65 – 1.51 (m, 1H); ^{13}**CNMR:** (101 MHz, Chloroform-d) δ 173.57, 145.89, 142.27, 137.40, 135.40, 128.42, 128.38, 127.76, 125.99, 121.20, 101.60, 81.05, 53.98, 51.61, 41.81, 37.40, 35.81, 33.94, 33.20, 18.72; **IR** (ATR, neat) 3061, 3027, 2922, 2853, 2191, 1731, 1679, 1603, 1435, 1407, 1179, 1155 cm^{-1}; **LRMS** (ESI + APCI) *m/z* [M+H] calcd 401.2, found 401.2

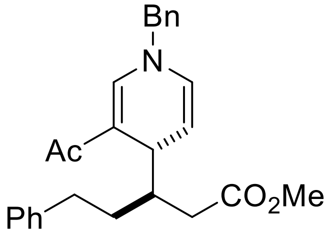

methyl 3-(3-acetyl-1-benzyl-1,4-dihydropyridin-4-yl)-5-phenylpentanoate (**3h**): Pale yellow oil. 46% yield, 4:1 rr, 5:1 dr, 50% ee. R_f = 0.2 (4:1 Hexanes:EtOAc); $[\alpha]_D^{21}$ = -52.7 (c = 0.008 g/mL); **HPLC analysis:** Chiralpak IA column, 93:7 hexanes/iso-propanol, 1.0 mL/min. Major: 71.8 min, minor: 78.2. ^{1}**HNMR**: (500 MHz,

Chloroform-d) δ 7.3a2 – 7.27 (m, 4H), 7.24 – 7.21 (m, 3H), 7.16 (d, J = 6.7 Hz, 4H), 5.98 (d, J = 7.8 Hz, 1H), 4.96 – 4.87 (m, 1H), 4.41 (s, 2H), 3.71 (s, 3H), 2.70 – 2.54 (m, 2H), 2.34 (d, J = 7.3 Hz, 2H), 2.19 (s, 4H), 2.10 (dd, J = 10.2, 6.0 Hz, 1H), 1.80 – 1.63 (m, 3H), 1.52 – 1.39 (m, 1H); **^{13}CNMR:** (126 MHz; CDCl3): δ 195.27, 173.97, 143.48, 142.86, 136.52, 129.20, 128.94, 128.39, 128.30, 128.00, 127.02, 125.61, 111.66, 106.15, 58.01, 51.55, 40.87, 36.00, 33.92, 32.64, 24.65; **IR** (ATR, neat) 3026, 2923, 2856, 1730, 1667, 1574, 1454, 1434, 1387, 1177 cm^{-1}. **LRMS** (ESI + APCI) *m/z* [M+H] calcd 404.2, found 404.3

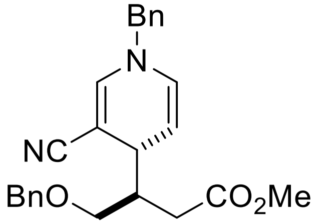

methyl 3-(1-benzyl-3-cyano-1,4-dihydropyridin-4-yl)-4-(benzyloxy)butanoate (**3i**): Pale yellow oil. 63% yield, 3:1 rr, 4:1 dr, 81% ee. R_f = 0.3 (4:1 Hexanes:EtOAc); $[\alpha]_D^{21}$ = -8.0 (c = 0.005 g/mL); **HPLC analysis:** Chiralpak IA column, 95:5 hexanes/iso-propanol, 1.0 mL/min. Major: 35.6 min, minor: 44.2 min. **^{1}HNMR**: (400 MHz, Chloroform-d) δ 7.33 (q, J = 4.1, 3.5 Hz, 9H), 7.24 – 7.21 (m, 2H), 7.18 – 7.15 (m, 2H), 6.71 (d, J = 1.6 Hz, 1H), 6.05 – 5.97 (m, 1H), 5.87 (d, J = 8.9 Hz, 1H), 4.62 (dd, J = 8.0, 4.6 Hz, 1H), 4.28 (s, 2H), 3.67 (s, 3H), 3.60 (s, 3H), 3.55 – 3.51 (m, 2H), 3.51 – 3.46 (m, 1H), 2.69 – 2.62 (m, 1H), 2.45 – 2.39 (m, 2H), 2.38 – 2.33 (m, 1H); **^{13}CNMR:** (101 MHz; CDCl3): 13C NMR (101 MHz, Chloroform-d) δ 173.31, 148.32, 144.17, 138.34, 135.97, 129.05, 128.26, 127.70, 127.22, 123.34, 121.40, 110.04, 104.09, 73.10, 70.42, 68.08, 57.89, 57.57, 56.76, 51.55, 42.64, 39.52, 34.24, 33.23, 31.23, 29.68; **IR** (ATR, neat) 3062, 3030, 2950, 2855, 2193, 1732, 1625, 1453, 1246, 1103 cm^{-1}; **LRMS** (ESI + APCI) *m/z* [M+H] calcd 403.2, found 403.2

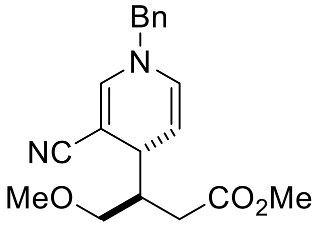

methyl 3-(1-benzyl-3-cyano-1,4-dihydropyridin-4-yl)-4-methoxybutanoate (**3j**): Pale yellow oil. 63% yield, 5:1 rr, 2:1 dr, 84% ee. R_f = 0.2 (4:1 Hexanes:EtOAc); $[\alpha]_D^{21}$ = -33.6 (c = 0.005 g/mL); **HPLC analysis:** Chiralpak IA column, 80:20 hexanes/iso-propanol, 1.0 mL/min. Major: 8.1 min, minor: 9.8 min. **1H NMR:** (400 MHz, Chloroform-d) δ 7.36 (dt, J = 13.0, 6.8 Hz, 5H), 7.18 (d, J = 6.6 Hz, 2H), 6.72 (d, J = 1.6 Hz, 1H), 5.89 (d, J = 8.0 Hz, 1H), 4.63 (dd, J = 8.1, 4.5 Hz, 1H), 4.30 (s, 2H), 3.69 (t, J = 4.2 Hz, 2H), 3.65 (s, 2H), 3.55 (dd, J = 9.6, 6.6 Hz, 1H), 3.52 – 3.48 (m, 1H), 3.37 (dd, J = 9.6, 7.4 Hz, 1H), 3.31 (s, 2H), 2.43 – 2.35 (m, 1H), 2.25 (dd, J = 6.9, 3.2 Hz, 1H); **13C NMR:** (101 MHz, Chloroform-d) δ 173.35, 148.35, 144.19, 135.98, 129.29, 129.05, 128.29, 127.23, 123.36, 121.37, 104.12, 79.78, 72.63, 58.77, 58.35, 57.60, 51.58, 42.59, 41.01, 39.46, 34.15, 33.03, 31.45, 31.14; **IR** (ATR, neat) 2950, 2925, 2192, 1730, 1672, 1589, 1413, 1179, 1118 cm^{-1} **LRMS** (ESI + APCI) *m/z* [M+H] calcd 327.2, found 327.2

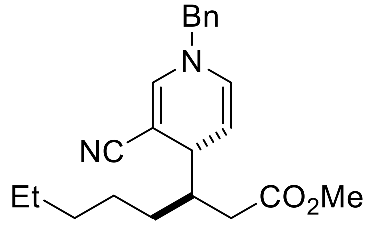

methyl 3-(1-benzyl-3-cyano-1,4-dihydropyridin-4-yl)octanoate (**3k**): Pale yellow oil. 63% yield, 4:1 rr, 3:1 dr, 84% ee. R_f = 0.2 (4:1 Hexanes:EtOAc); $[\alpha]_D^{21}$ = -23.7 (c = 0.01 g/mL); **HPLC analysis:** Chiralpak IA column, 90:10 hexanes/iso-propanol, 1.0 mL/min. Major: 6.4 min, minor: 5.8 min. **^{1}HNMR**: (400 MHz, Chloroform-d) δ 7.40 – 7.29 (m, 3H), 7.22 – 7.16 (m, 2H), 6.70 (d, J = 1.6 Hz, 1H), 5.87 (d, J = 8.8 Hz, 1H), 4.60 (dd, J = 8.2, 4.4 Hz, 1H), 4.28 (s, 2H), 3.65 (s, 3H), 3.38 (t, J = 3.7 Hz, 1H), 2.30 (dd, J = 7.2, 2.9 Hz, 2H), 1.99 – 1.91 (m, 1H), 1.69 – 1.60 (m, 1H), 1.38 – 1.16 (m, 9H), 0.92 – 0.85 (m, 4H); **^{13}CNMR:** (101 MHz, Chloroform-d) δ 173.70, 161.51, 144.08, 136.18, 128.99, 128.19, 127.16, 122.89, 121.22, 110.47, 103.75, 102.63, 92.89, 80.85, 57.52, 55.30, 51.53, 41.78, 36.39, 35.60, 33.30, 32.01, 30.77, 28.49, 27.00, 22.55, 14.06; **IR** (ATR, neat) 2952, 2927, 2856, 2192, 1731, 1673, 1591, 1411, 1204, 1151 cm^{-1}; **LRMS** (ESI + APCI) *m/z* [M+H] calcd 353.2, found 353.3

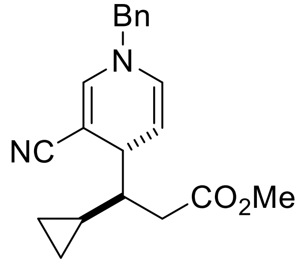

methyl 3-(1-benzyl-3-cyano-1,4-dihydropyridin-4-yl)-3-cyclopropylpropanoate (**3l**): Pale yellow oil. 31% yield, 3:1 rr, 3:1 dr, 85% ee. R_f = 0.4 (4:1 Hexanes:EtOAc); $[\alpha]_D^{21}$ = -60.0 (c = 0.01 g/mL); **HPLC analysis:** Chiralpak IA column, 90:10 hexanes/iso-propanol, 1.0 mL/min. Major: 14.3 min, minor: 16.2 min. **^{1}HNMR**: (400 MHz, Chloroform-d) δ 7.35 (d, J = 7.1 Hz, 4H), 7.21 – 7.17 (m, 2H), 6.71 (d, J = 1.6 Hz, 1H), 5.91 (d, J = 10.2 Hz, 1H), 4.62 (dd, J = 8.1, 4.6 Hz, 1H), 4.29 (s, 3H), 3.66 – 3.64 (m, 3H), 3.46 – 3.41 (m, 1H), 2.42 (dd, J = 24.9, 7.4 Hz, 3H), 1.24 – 1.17 (m, 1H), 0.94 – 0.82 (m, 2H), 0.50 – 0.47 (m, 1H), 0.45 – 0.41 (m, 1H), 0.32 (dt, J = 9.0, 4.5 Hz, 1H), 0.08 (tt, J = 9.5, 4.6 Hz, 2H); **^{13}CNMR:** (101 MHz; CDCl3): δ 173.59, 148.79, 144.05, 136.16, 128.98, 127.19, 123.45, 121.54, 110.13, 81.01, 59.04, 47.88, 45.35, 37.85, 36.50, 34.53, 13.18, 11.62, 4.58, 3.57; **IR** (ATR, neat) 3064, 3002, 2923, 2851, 2192, 1673, 1591, 1437, 1414, 1245, 1180 cm^{-1} **LRMS** (ESI + APCI) *m/z* [M+H] calcd 323.2, found 323.2

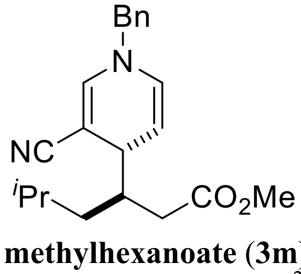

methyl 3-(1-benzyl-3-cyano-1,4-dihydropyridin-4-yl)-5-methylhexanoate (**3m**): Pale yellow oil. 52% yield, 3:1 rr, 2:1 dr, 58% ee. R_f = 0.2 (4:1 Hexanes:EtOAc); $[\alpha]_D^{21}$ = -18.9 (c = 0.010 g/mL); **HPLC analysis:** Chiralpak IE column, 90:10 hexanes/iso-propanol, 1.0 mL/min. Major: 38.1 min, minor: 33.8 min. **^{1}HNMR**: (400 MHz,

Chloroform-d) δ 7.35 (dq, J = 11.9, 6.6, 5.2 Hz, 4H), 7.21 – 7.19 (m, 1H), 6.71 (d, J = 1.6 Hz, 1H), 5.88 (d, J = 8.2 Hz, 1H), 4.60 (dd, J = 8.1, 4.3 Hz, 1H), 4.28 (s, 2H), 3.66 (d, J = 6.9 Hz, 3H), 3.38 (s, 1H), 2.28 (t, J = 6.9 Hz, 2H), 1.60 (ddd, J = 19.8, 14.2, 6.6 Hz, 2H), 1.49 – 1.43 (m, 1H), 1.18 – 1.07 (m, 1H), 0.91 (d, J = 6.5 Hz, 6H); **^{13}CNMR:** (101 MHz, Chloroform-d) δ 173.60, 161.51, 148.80, 136.20, 129.59, 129.42, 128.99, 128.20, 127.22, 127.14, 122.98, 121.13, 103.61, 102.61, 92.90, 57.53, 55.31, 51.53, 40.04, 39.21, 36.33, 35.74, 25.19, 23.30, 22.10; **IR** (ATR, neat) 2926, 2867, 2193, 1733, 1674, 1593, 1454, 1206, 1194, 1152 cm^{-1} **LRMS** (ESI + APCI) *m/z* [M+H] calcd 339.2, found 339.2

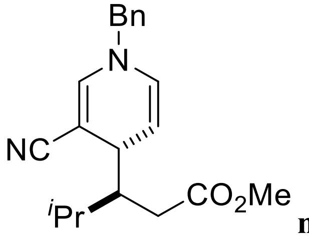

methyl (S)-3-((S)-1-benzyl-3-cyano-1,4-dihydropyridin-4-yl)-4-methylpentanoate (**3n**): Pale yellow oil. 54% yield, 5:1 rr, 3:1 dr, 42% ee. R_f = 0.2 (4:1 Hexanes:EtOAc); $[\alpha]_D^{21}$ = 0.5699 (c = 0.011 g/mL); **HPLC analysis:** Chiralpak IA column, 90:10 hexanes/iso-propanol, 1.0 mL/min. Major: 14.6 min, minor: 10.2 min. **^{1}HNMR**: (500 MHz, Chloroform-d) δ 7.46 – 7.32 (m, 8H), 7.23 (q, J = 8.2, 7.0 Hz, 4H), 6.67 (d, J = 1.5 Hz, 1H), 5.91 – 5.85 (m, 1H), 4.59 (dd, J = 8.2, 4.3 Hz, 1H), 4.31 (d, J = 3.9 Hz, 3H), 3.68 (s, 3H), 3.54 (t, J = 3.7 Hz, 1H), 3.36 (d, J = 5.9 Hz, 1H), 2.60 (dd, J = 15.8, 5.8 Hz, 1H), 2.37 – 2.31 (m, 1H), 1.94 (qd, J = 7.1, 3.2 Hz, 1H), 1.77 – 1.69 (m, 2H), 0.96 (dd, J = 6.6, 5.1 Hz, 8H); **^{13}CNMR:** (126 MHz, Chloroform-d) δ 174.40, 143.73, 136.06, 129.61, 129.03, 128.24, 127.92, 127.30, 120.88, 102.91, 82.59, 57.44, 51.56, 47.66, 35.24, 34.24, 28.80, 20.59; **IR** (ATR, neat) 2958, 2192, 1732, 1672, 1627, 1592, 1367, 1413 1181, 1118, 703 cm^{-1} **LRMS** (ESI + APCI) *m/z* [M+H] calcd 325.4, found 325.1

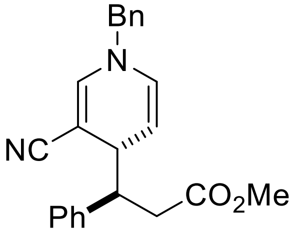

methyl (R)-3-((S)-1-benzyl-3-cyano-1,4-dihydropyridin-4-yl)-3-phenylpropanoate (**3o**): Pale yellow oil. 40.5 mg, 57% yield, 8:1 rr, 1:1 dr, 24% ee. R_f = 0.2 (4:1 Hexanes:EtOAc); $[\alpha]_D^{21}$ = -20.0 (c = 0.017 g/mL); **HPLC analysis:** Chiralpak IE column, 70:30 hexanes/iso-propanol, 1.0 mL/min. Major: 14.3 min, minor: 16.2 min. **^{1}HNMR**: (500 MHz, Chloroform-d) δ 7.42 – 7.29 (m, 12H), 7.28 – 7.24 (m, 3H), 7.21 (d, J = 7.2 Hz, 2H), 7.09 (d, J = 6.9 Hz, 2H), 6.82 (dd, J = 6.5, 2.8 Hz, 2H), 6.68 (d, J = 1.5 Hz, 1H), 6.48 (d, J = 1.5 Hz, 1H), 5.88 (d, J = 8.1 Hz, 1H), 5.78 (d, J = 8.1 Hz, 1H), 4.70 (dd, J = 8.1, 4.7 Hz, 1H), 4.54 (dd, J = 8.1, 4.5 Hz, 1H), 4.26 (s, 2H), 4.12 (s, 2H), 3.61 (dd, J = 6.4, 3.8 Hz, 1H), 3.59 (s, 3H), 3.59 (s, 3H), 3.55 (t, J = 4.1 Hz, 1H), 3.43 – 3.35 (m, 2H), 3.00 – 2.74 (m, 4H); **^{13}CNMR:** (126 MHz, Chloroform-d) δ 172.91, 172.35, 144.05, 143.71, 139.89, 139.52, 129.76, 129.56, 129.17, 129.06, 128.93, 128.35, 128.26, 128.24, 127.97, 127.95, 127.17, 126.88, 126.80, 121.28, 120.99, 81.11, 57.47, 57.21, 51.70, 51.66, 47.68, 40.22, 39.14, 35.88, 34.92; **IR** (ATR, neat) 3029, 2949,

2910, 2191, 1733, 1673, 1591, 1453, 1413, 1184, 1160, 740, 702 cm^{-1} **LRMS** (ESI) *m/z* [M+H] calcd 359.2, found 359.2

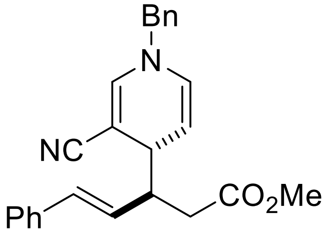

methyl (S,E)-3-((S)-1-benzyl-3-cyano-1,4-dihydropyridin-4-yl)-5-phenylpent-4-enoate (**3p**): Pale yellow oil. 38% yield, 5:1 rr, 1:1 dr, 24% ee. R_f = 0.2 (4:1 Hexanes:EtOAc); $[\alpha]_D^{21}$ = -91.8 (c = 0.006 g/mL); **HPLC analysis:** Chiralpak IE column, 80:20 hexanes/iso-propanol, 1.0 mL/min. Major: 36.4 min, minor: 32.9 min. ^{1}**HNMR**: (500 MHz, Chloroform-d) δ 7.36 (dd, J = 12.3, 7.8 Hz, 4H), 7.34 – 7.30 (m, 4H), 7.26 (dd, J = 7.6, 3.4 Hz, 2H), 7.21 (d, J = 7.8 Hz, 1H), 7.09 (dd, J = 7.5, 2.7 Hz, 2H), 6.68 (t, J = 1.8 Hz, 1H), 6.18 (dd, J = 15.8, 9.4 Hz, 1H), 5.94 (dt, J = 8.2, 2.2 Hz, 1H), 4.77 – 4.68 (m, 1H), 4.27 (s, 2H), 3.66 (s, 3H), 3.59 (s, 1H), 3.50 (dt, J = 12.8, 4.1 Hz, 1H), 2.90 (dt, J = 10.2, 4.7 Hz, 1H), 2.84 – 2.73 (m, 1H), 2.60 – 2.49 (m, 1H).^{13}**CNMR:** (126 MHz, Chloroform-d) δ 172.46, 143.83, 133.09, 132.32, 129.95, 129.08, 129.02, 128.57, 128.28, 127.36, 127.19, 127.05, 126.50, 103.25, 102.21, 57.53, 51.74, 47.70, 40.23, 39.16, 38.78, 38.45, 35.91; **IR** (ATR, neat) 3026, 2954, 2923, 2856, 2191, 1732, 1672, 1590, 1412, 1182, 747, 701 cm^{-1} **LRMS** (ESI) *m/z* [M+H] calcd 385.2, found 385.

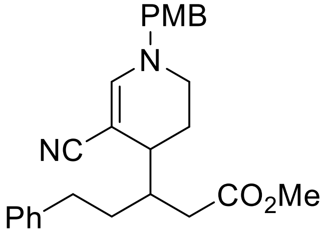

methyl 3-(5-cyano-1-(4-methoxybenzyl)-1,2,3,4-tetrahydropyridin-4-yl)-5-phenylpentanoate (**9**): Palladium hydroxide on carbon (20 wt%, 14.0 mg, 0.02 mmol) was added to a vial containing dihydropyridine **3i** (42.3 mg, 0.1 mmol) and methanol (2 mL) at room temperature under argon. The vial was then placed under vacuum and back-filled with Argon two times. After removal of the argon atmosphere a third time with vacuum, a hydrogen balloon was placed in the vial and the reaction stirred was stirred for 12 h. After this time, celite is added to the reaction mixture, and the solids were removed by filtration through celite to give 37.4 mg (88%, 3:1 dr, 86% ee) of a yellow oil. R_f = 0.2 (4:1 Hexanes:EtOAc); $[\alpha]_D^{21}$ = -35.2 (c = 0.125 g/mL); **HPLC analysis:** Chiralpak IB column, 85:15 hexanes/iso-propanol, 1.0 mL/min. Major: 31.2 min, minor: 29.0 min. ^{1}**HNMR**: (500 MHz, Chloroform-d) δ 7.32 – 7.27 (m, 2H), 7.23 – 7.17 (m, 3H), 7.14 – 7.09 (m, 2H), 6.98 (dd, J = 11.3, 1.2 Hz, 1H), 6.93 – 6.87 (m, 2H), 4.17 (d, J = 7.4 Hz, 2H), 3.83 (s, 3H), 3.71 (s, 3H), 3.01 (dt, J = 12.3, 5.5 Hz, 2H), 2.74 – 2.63 (m, 2H), 2.57 (dd, J = 15.2, 6.3 Hz, 1H), 2.47 (d, J = 7.9 Hz, 1H), 2.34 – 2.21 (m, 1H); ^{13}**CNMR:** (101 MHz, Chloroform-d) δ 173.41, 159.47, 142.24, 128.91, 128.38, 125.84, 122.88, 114.29, 58.95, 55.33, 51.65, 43.85, 38.51, 37.83, 36.73, 35.86, 34.48, 33.98, 29.70, 22.53; **IR** (ATR, neat) 2927, 2855, 2180, 1732, 1617, 1512, 1248, 1175, 1031 cm^{-1} **LRMS** (ESI) *m/z* [M+H] calcd 419.2, found 419.2

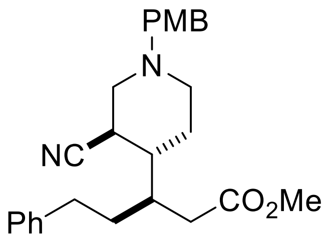

methyl (R)-3-((3R,4R)-3-cyano-1-(4-methoxybenzyl)piperidin-4-yl)-5-phenylpentanoate (**10**): A solution of dihydropyridine **3i** (44.3 mg, 0.11 mmol) in CH_2Cl_2 (2 mL) was cooled to -10℃, and triethylsilane (340 uL, 20 equiv) was added along with trifluoracetic acid (204 uL, 25 equiv). This mixture was then allowed to stir while warming to room temperature. After 12 h, volatiles were removed under reduced pressure. The resulting crude oil was purified by column chromatography (DCM/MeOH) to give **10** (51%, 22.8 mg, 0.054 mmol, 3:1 dr, 84% ee) as an off-white solid. R_f = 0.2 (98:2 DCM:MeOH); $[\alpha]_D^{21}$ = -37.8 (c = 0.008 g/mL); **HPLC analysis:** Chiralpak IB column, 85:15 hexanes/iso-propanol, 1.0 mL/min. Major: 10.6 min, minor: 11.6 min. **^{1}HNMR**: (500 MHz, Chloroform-d) δ 7.34 – 7.26 (m, 4H), 7.22 (d, J = 7.3 Hz, 1H), 7.18 (d, J = 7.2 Hz, 2H), 6.96 (d, J = 8.4 Hz, 2H), 4.11 (dd, J = 64.4, 13.0 Hz, 2H), 3.85 (s, 3H), 3.70 (s, 3H), 3.57 (d, J = 11.6 Hz, 1H), 3.48 – 3.38 (m, 1H), 2.80 – 2.66 (m, 2H), 2.60 (dt, J = 15.2, 7.5 Hz, 3H), 2.44 – 2.31 (m, 1H), 2.25 (dd, J = 15.2, 9.9 Hz, 1H), 1.89 (dd, J = 26.0, 10.7 Hz, 3H), 1.81 – 1.71 (m, 1H), 1.42 (dtd, J = 14.4, 9.5, 5.2 Hz, 1H); **^{13}CNMR:** (126 MHz, Chloroform-d) δ 172.46, 140.59, 128.60, 128.25, 126.26, 119.17, 116.76, 114.90, 60.42, 55.43, 51.92, 51.70, 51.51, 39.71, 36.06, 35.90, 33.72, 30.50, 29.07, 21.82; **IR** (ATR, neat) 2925, 1734, 1673, 1612, 1514, 1454, 1251, 1180, 1032, 830 cm^{-1} **LRMS** (ESI) *m/z* [M+H] calcd 421.2, found 421.3

Proposed stereochemical model to account for selectivity:

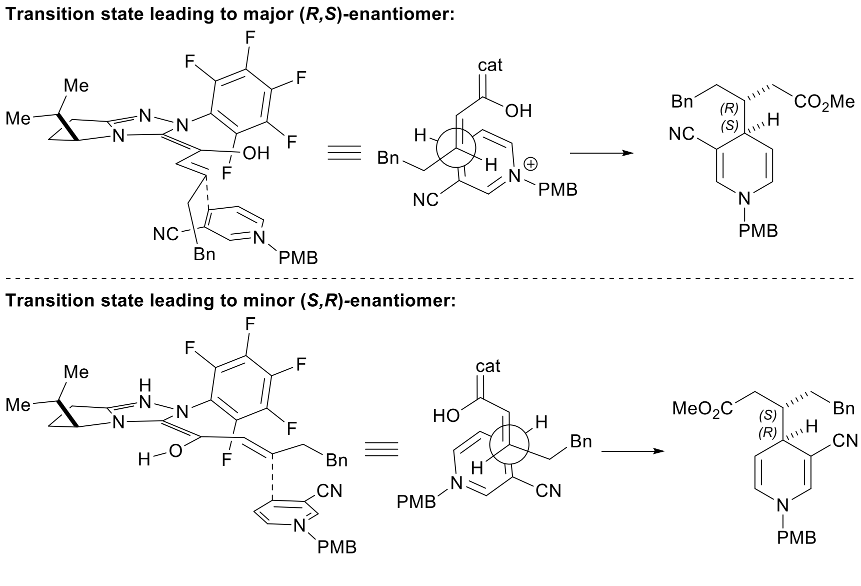

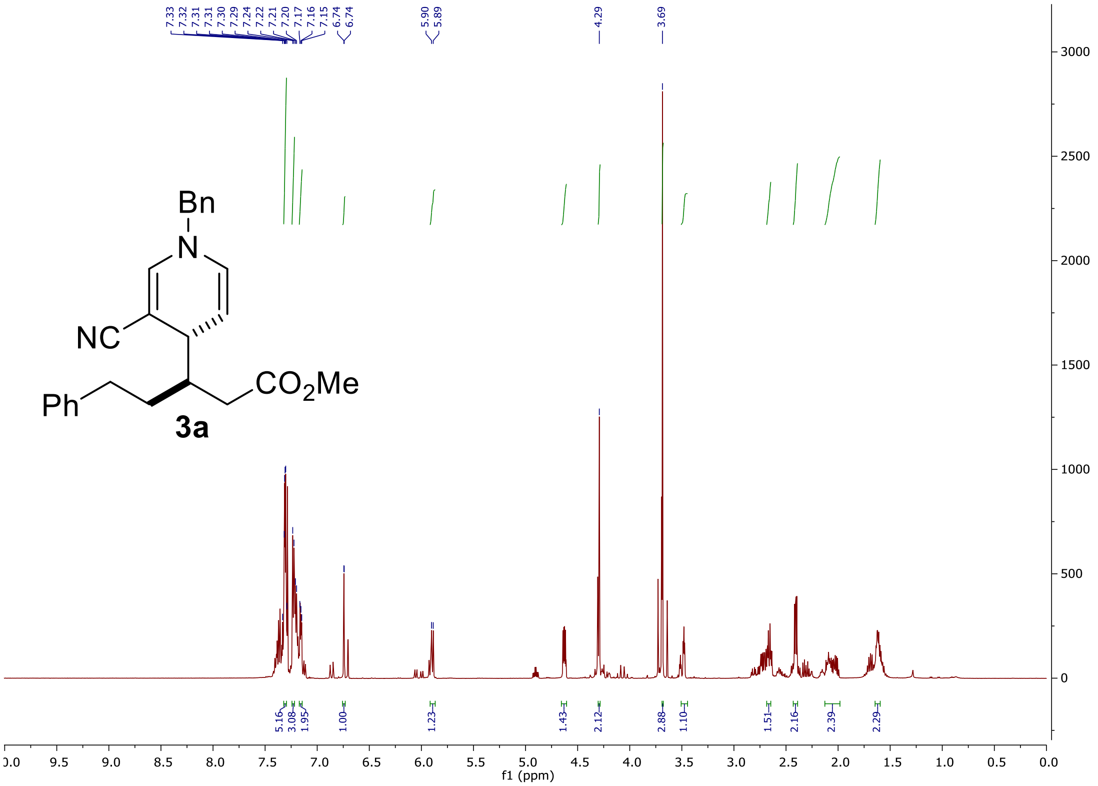
Bn
N
NC
Ph
CO2Me
3a
f1 (ppm)

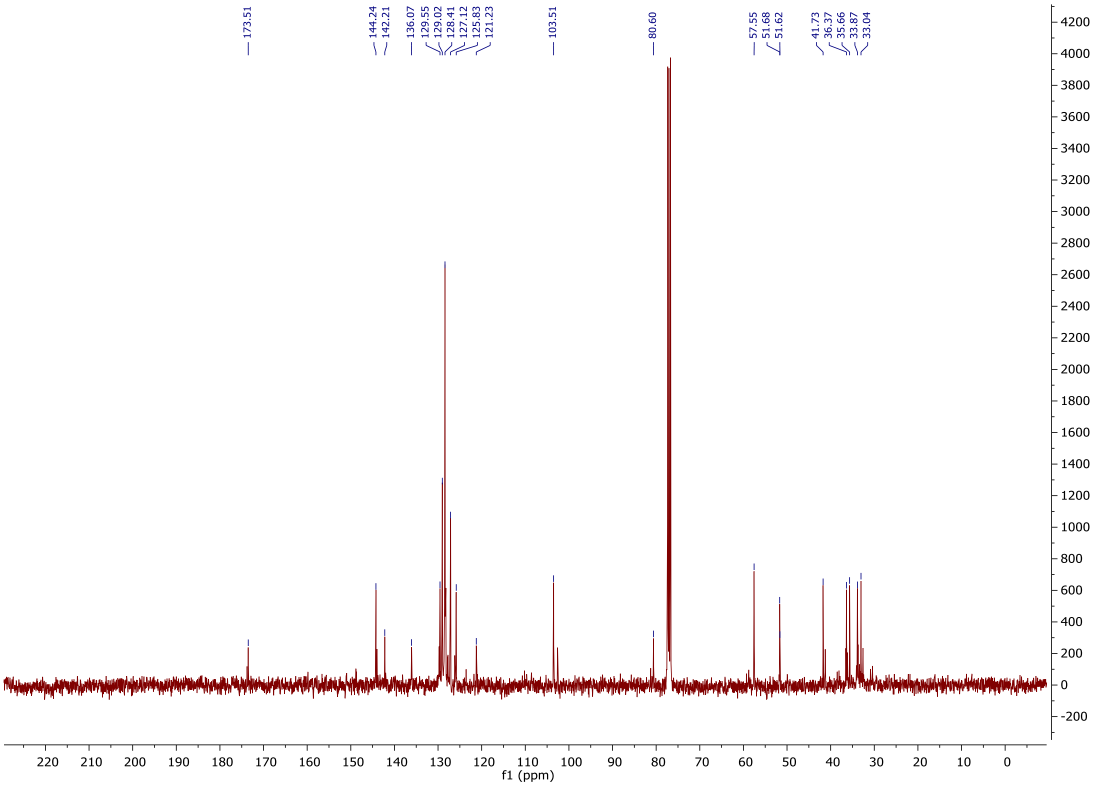
f1 (ppm)

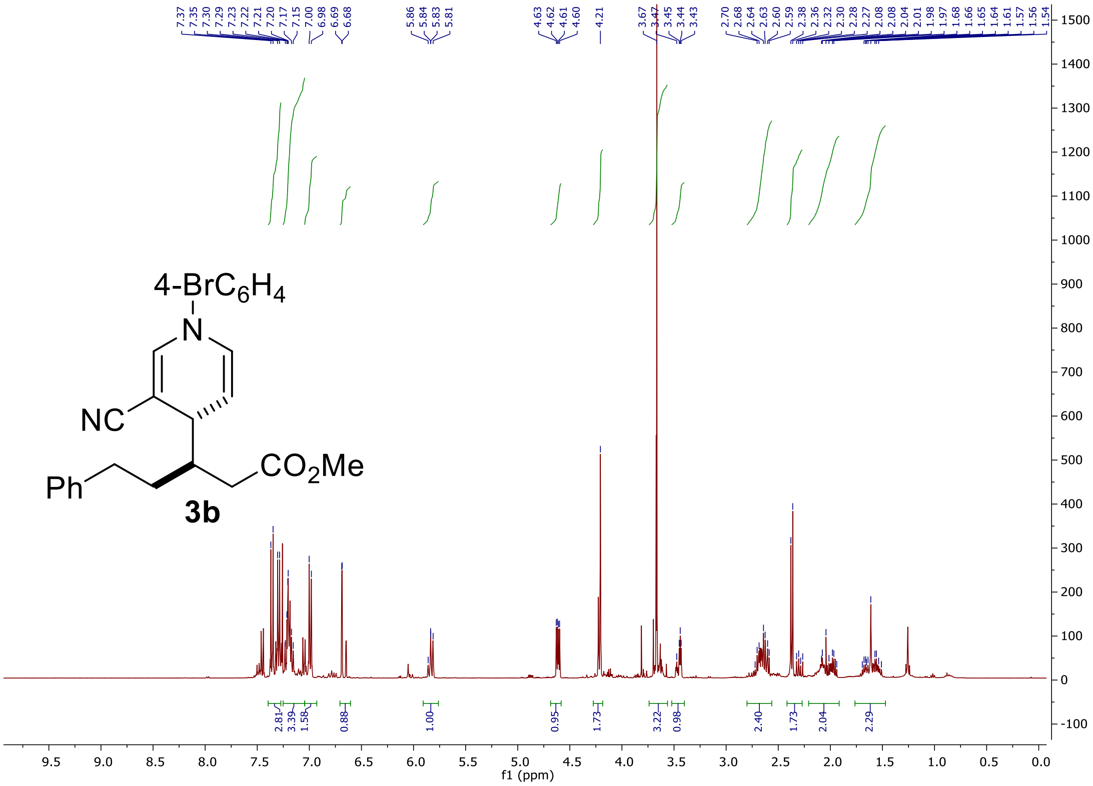
7.37 7.35 7.30 7.29 7.23 7.22 7.21 7.20 7.17 7.15 7.00 6.98 6.69 6.68
5.86 5.84 5.83 5.81
4.63 4.62 4.61 4.60 4.21
3.67 3.47 3.45 3.44 3.43
2.70 2.68 2.64 2.63 2.60 2.59 2.38 2.36 2.32 2.30 2.28 2.27 2.08 2.08 2.04 2.01 1.98 1.97 1.68 1.66 1.65 1.64 1.61 1.57 1.56 1.54
4-BrC6H4
N
NC
CO2Me
Ph
3b
2.81 3.39 1.58 0.88 1.00 0.95 1.73 3.22 0.98 2.40 1.73 2.04 2.29
f1 (ppm)

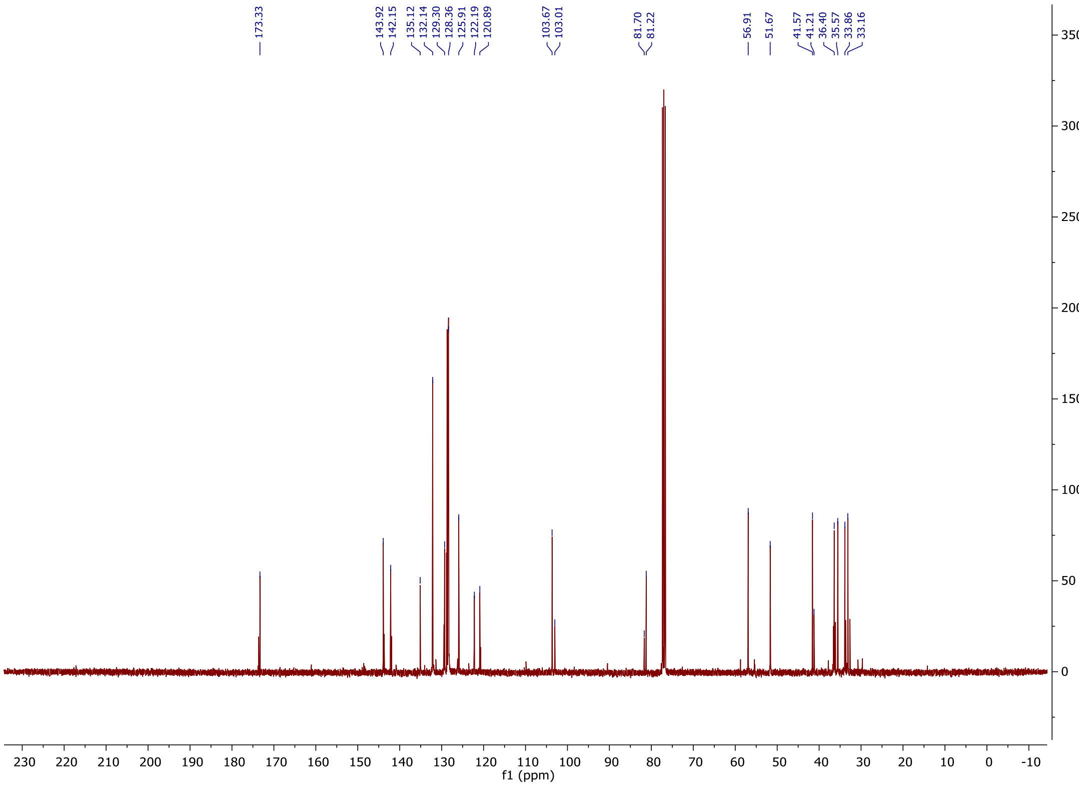
173.33
143.92 142.15 135.12 132.14 129.30 128.36 125.91 122.19 120.89
103.67 103.01
81.70 81.22
56.91 51.67 41.57 41.21 36.40 35.57 33.86 33.16
f1 (ppm)

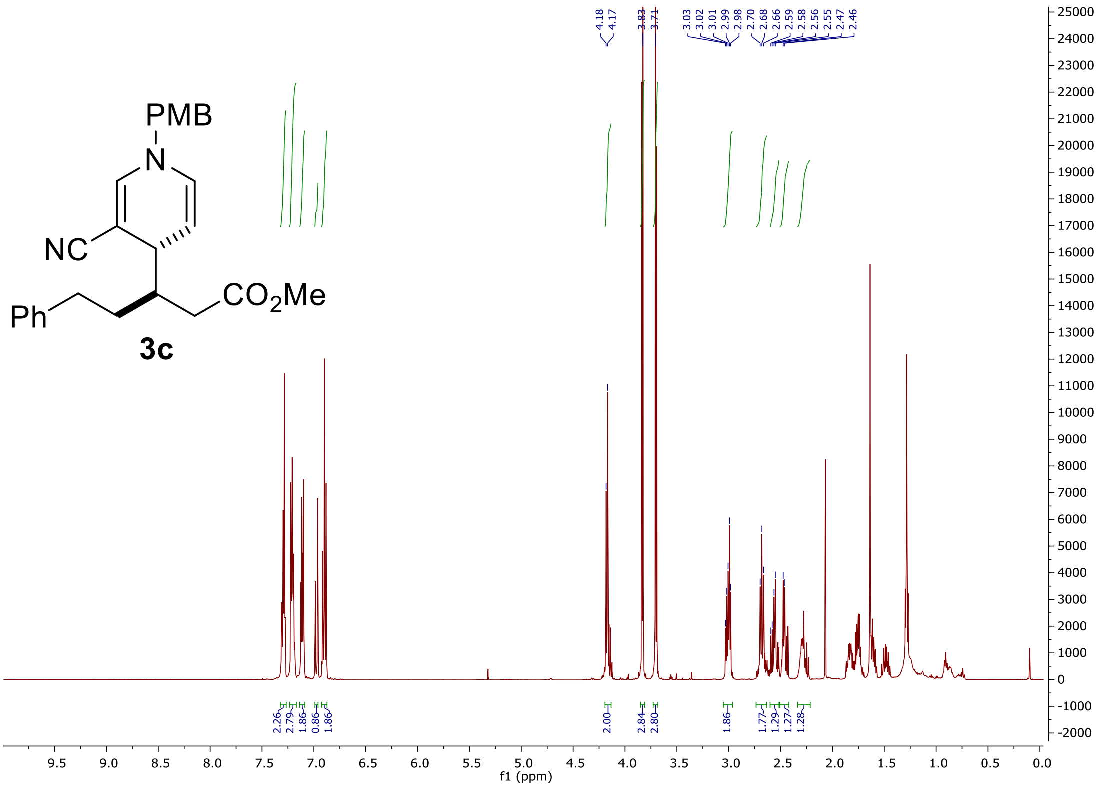
PMB
N
NC
CO2Me
Ph
3c
4.18
4.17
3.83
3.71
3.03
3.02
3.01
2.99
2.98
2.70
2.68
2.66
2.59
2.58
2.56
2.55
2.47
2.46
2.26
2.79
1.86
0.86
1.86
2.00
2.84
2.80
1.86
1.77
1.29
1.27
1.28
f1 (ppm)

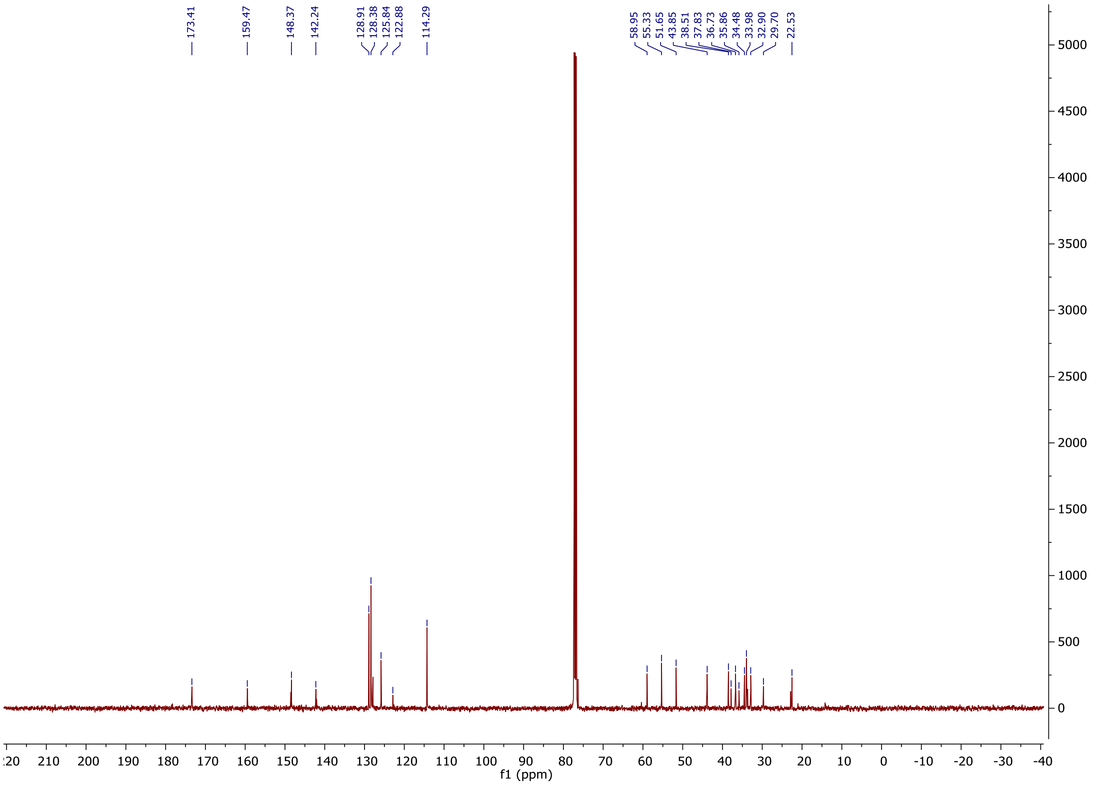
173.41
159.47
148.37
142.24
128.91
128.38
125.84
122.88
114.29
58.95
55.33
51.65
43.85
38.51
37.83
36.73
35.86
34.48
33.98
32.90
29.70
22.53
f1 (ppm)

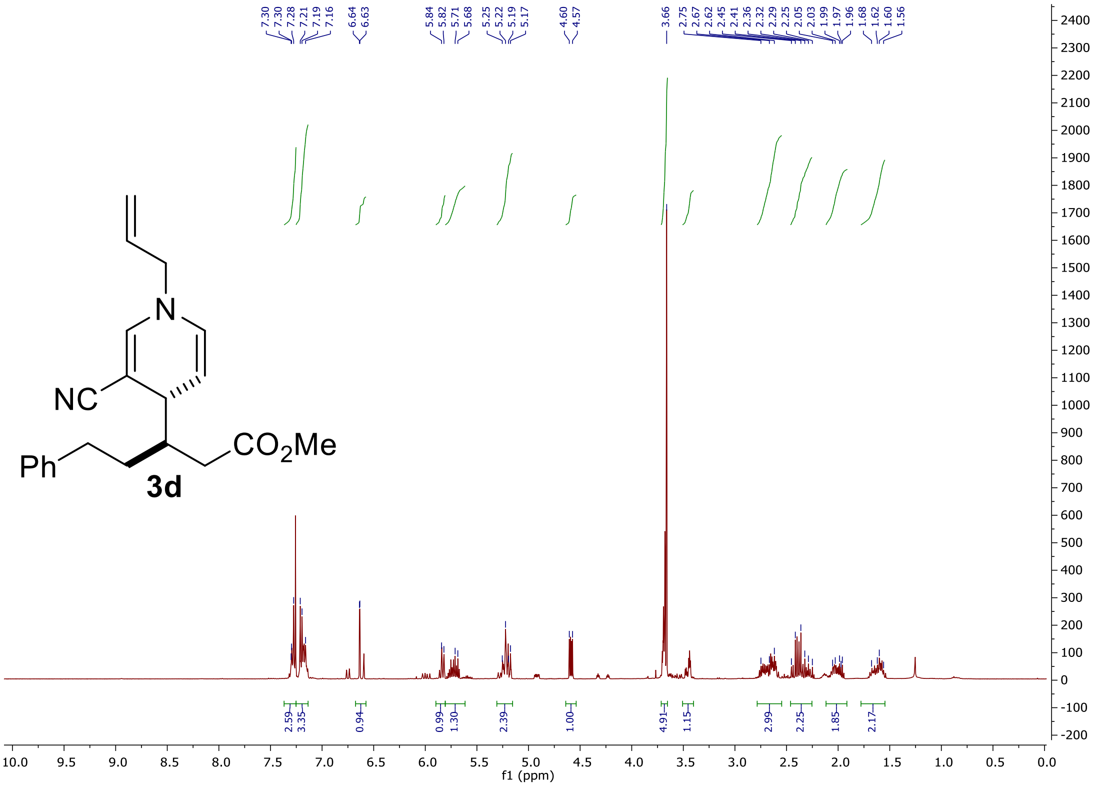
7.30 7.30 7.28 7.21 7.19 7.16 6.64 6.63 5.84 5.82 5.71 5.68 5.25 5.22 5.19 5.17 4.60 4.57 3.66 2.75 2.67 2.62 2.45 2.41 2.36 2.32 2.29 2.25 2.05 2.03 1.99 1.97 1.96 1.68 1.62 1.60 1.56
NC
N
CO_2Me
Ph
3d
2.59 3.35 0.94 0.99 1.30 2.39 1.00 4.91 1.15 2.99 2.25 1.85 2.17
f1 (ppm)

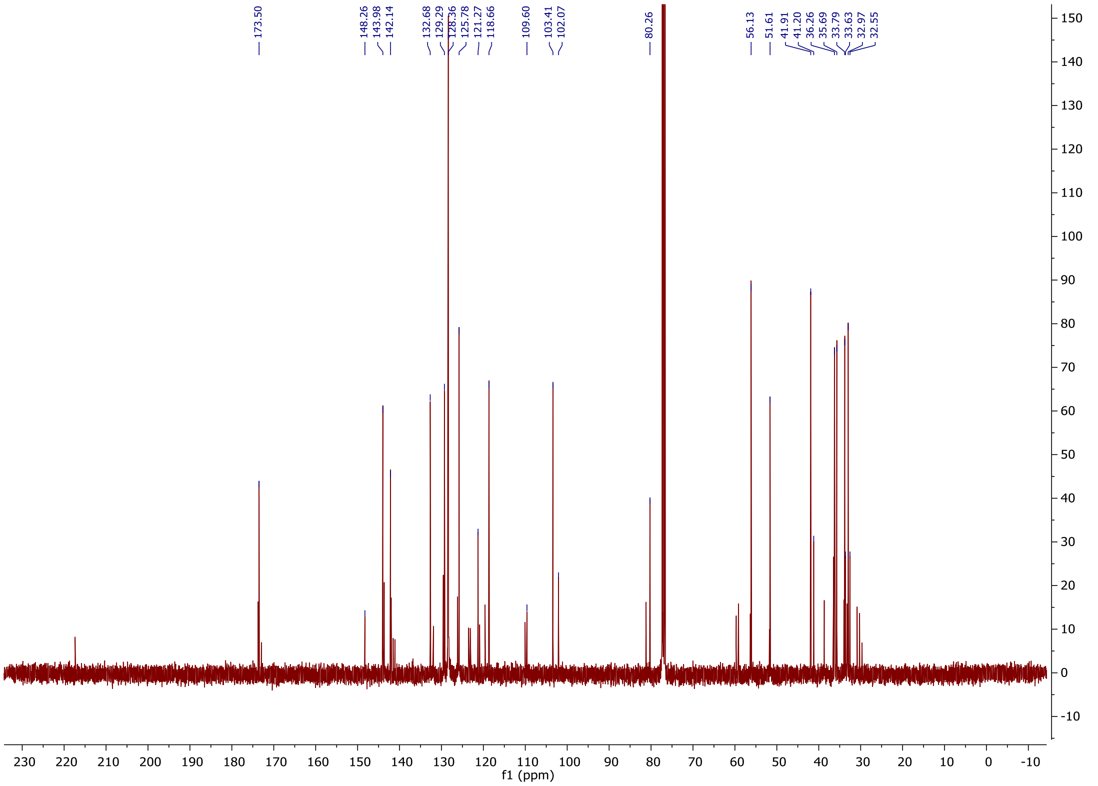
173.50 148.26 143.98 142.14 132.68 129.29 128.36 125.78 121.27 118.66 109.60 103.41 102.07 80.26 56.13 51.61 41.91 41.20 36.26 35.69 33.79 33.63 32.97 32.55
f1 (ppm)

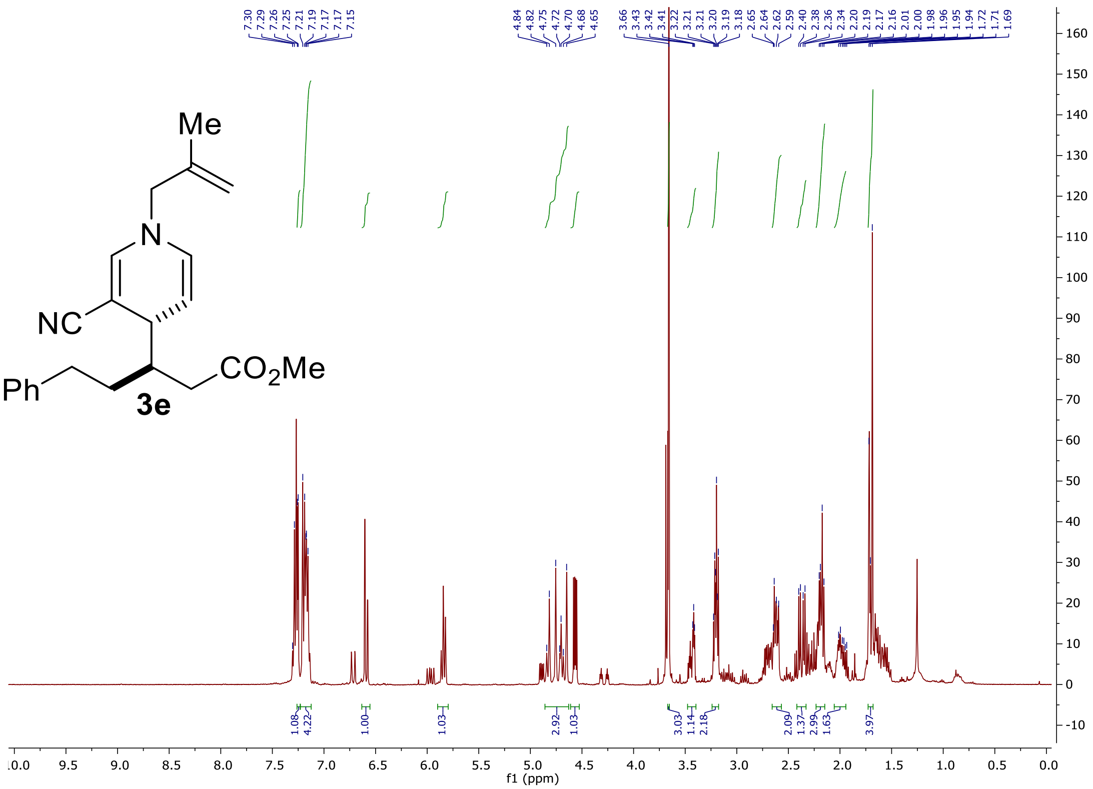
7.30
7.29
7.26
7.25
7.21
7.19
7.17
7.17
7.15
4.84
4.82
4.75
4.72
4.70
4.68
4.65
3.66
3.43
3.42
3.41
3.22
3.21
3.21
3.20
3.19
3.18
2.65
2.64
2.62
2.59
2.40
2.38
2.36
2.34
2.20
2.19
2.17
2.16
2.01
2.00
1.98
1.96
1.95
1.94
1.72
1.71
1.69
Me
N
NC
CO2Me
Ph
3e
1.08
4.22
1.00
1.03
2.92
1.03
3.03
1.14
2.18
2.09
1.37
2.99
1.63
3.97
f1 (ppm)

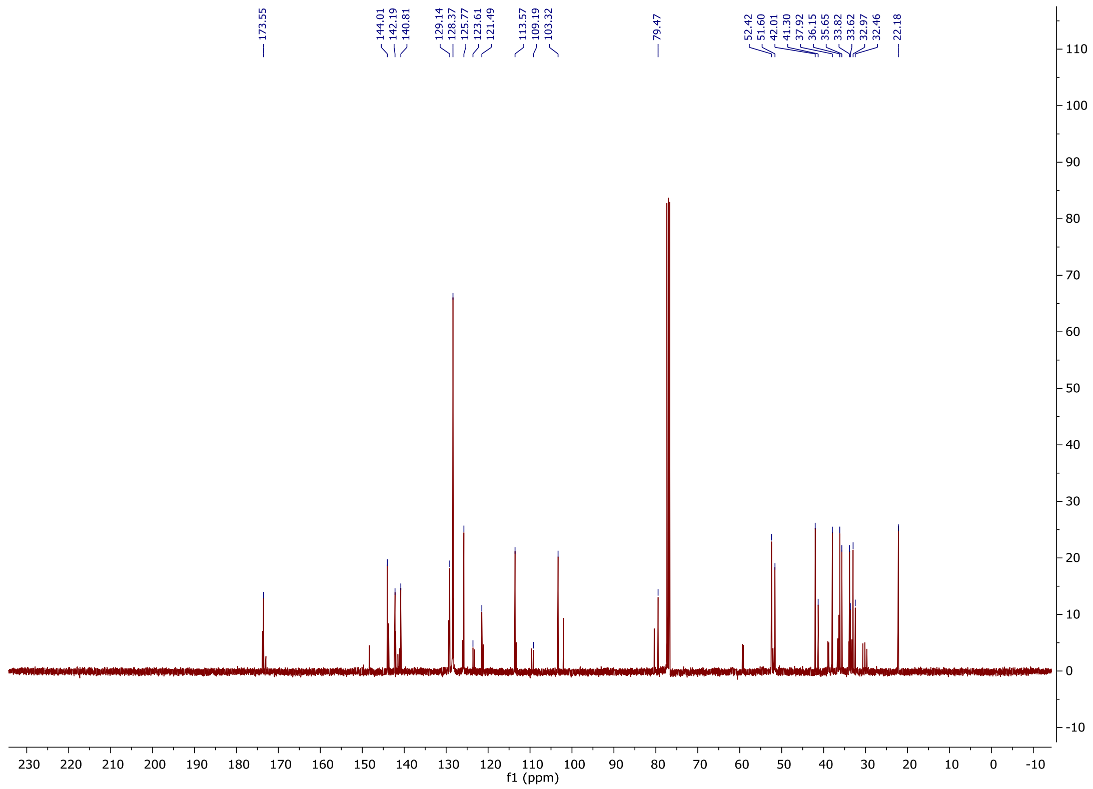
173.55
144.01
142.19
140.81
129.14
128.37
125.77
123.61
121.49
113.57
109.19
103.32
79.47
52.42
51.60
42.01
41.30
37.92
36.15
35.65
33.82
33.62
32.97
32.46
22.18
f1 (ppm)

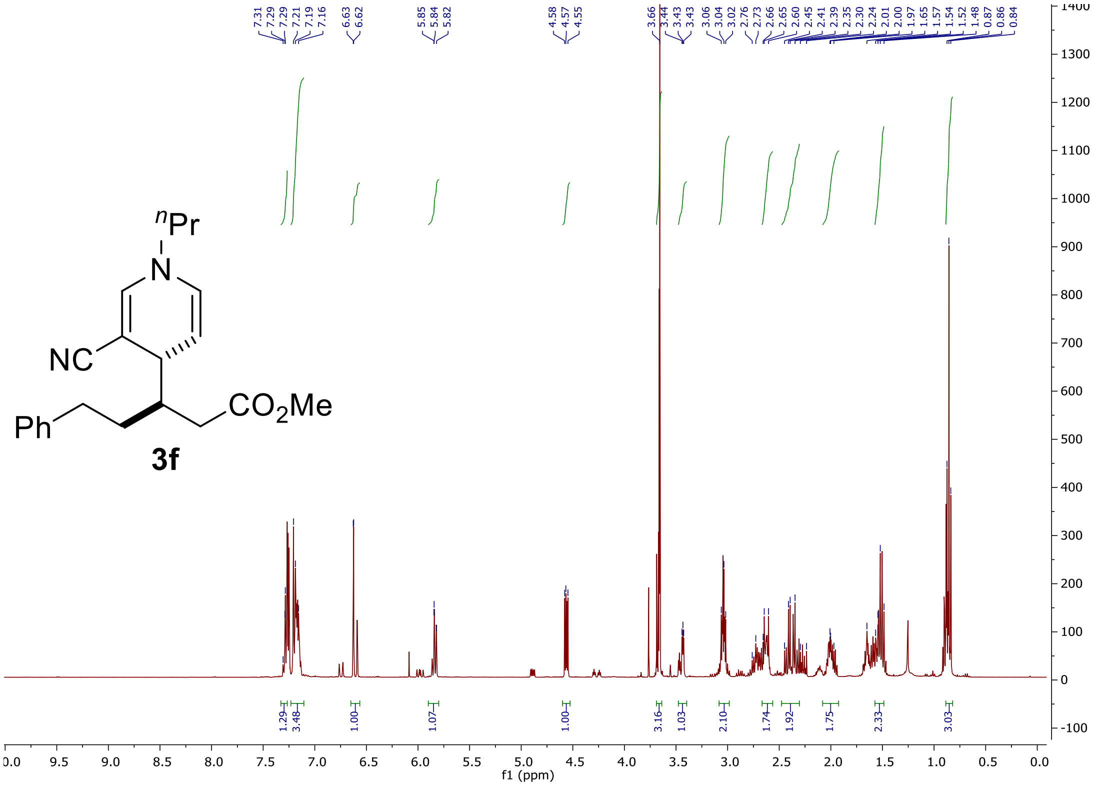
nPr
N
NC
Ph
CO2Me
3f
f1 (ppm)

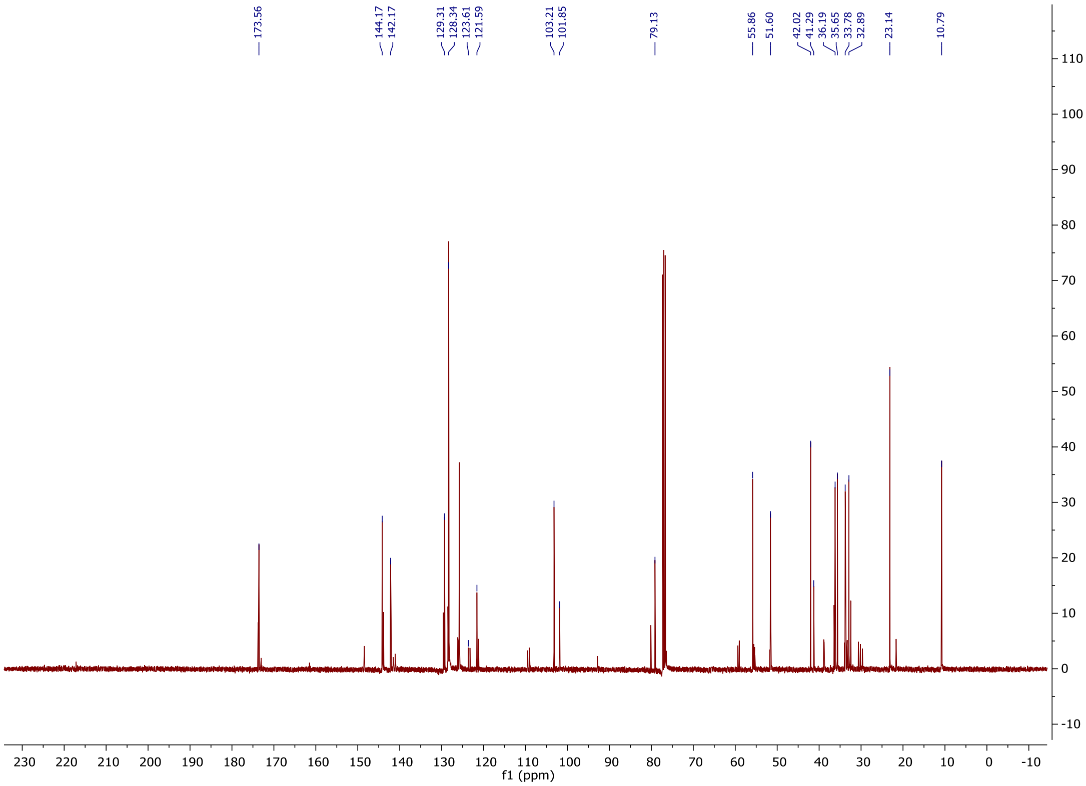
f1 (ppm)

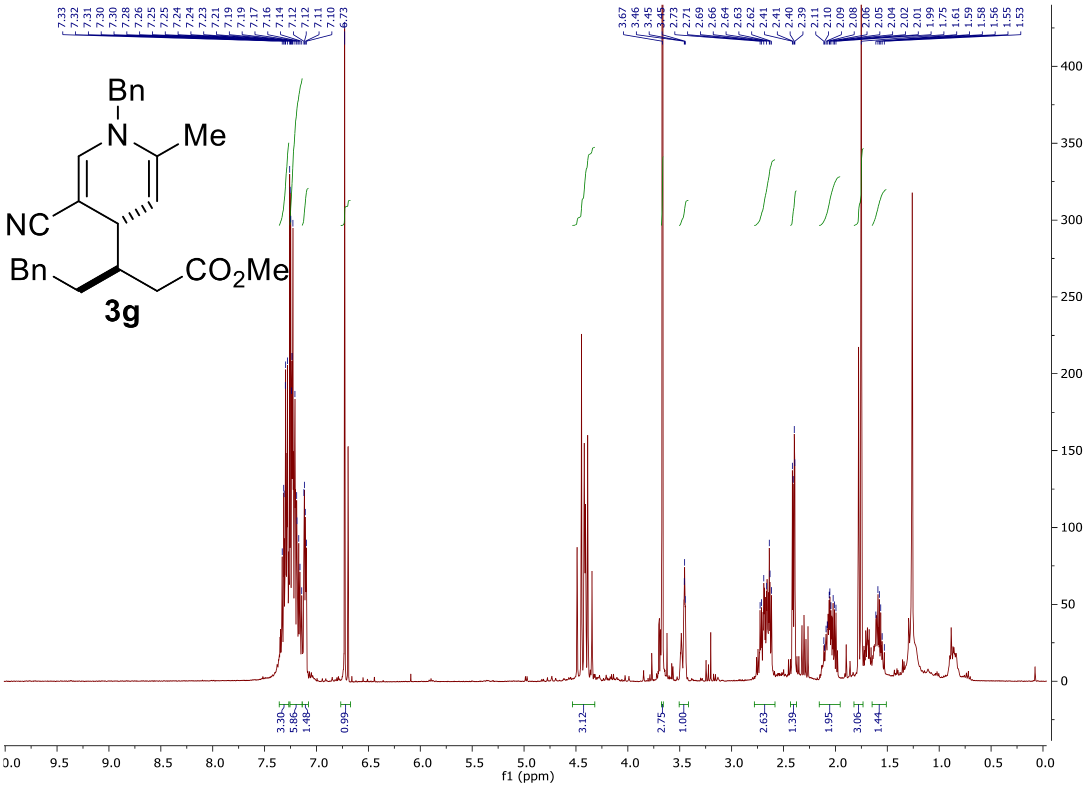
Bn
N
Me
NC
Bn
CO2Me
3g
7.33 7.32 7.31 7.30 7.30 7.28 7.26 7.25 7.25 7.24 7.24 7.23 7.21 7.19 7.19 7.17 7.16 7.14 7.12 7.12 7.11 7.10 6.73
3.67 3.46 3.45 3.45 2.73 2.71 2.69 2.66 2.64 2.63 2.62 2.41 2.41 2.40 2.39 2.11 2.10 2.09 2.08 2.06 2.05 2.04 2.02 2.01 1.99 1.75 1.61 1.59 1.58 1.56 1.55 1.53
f1 (ppm)

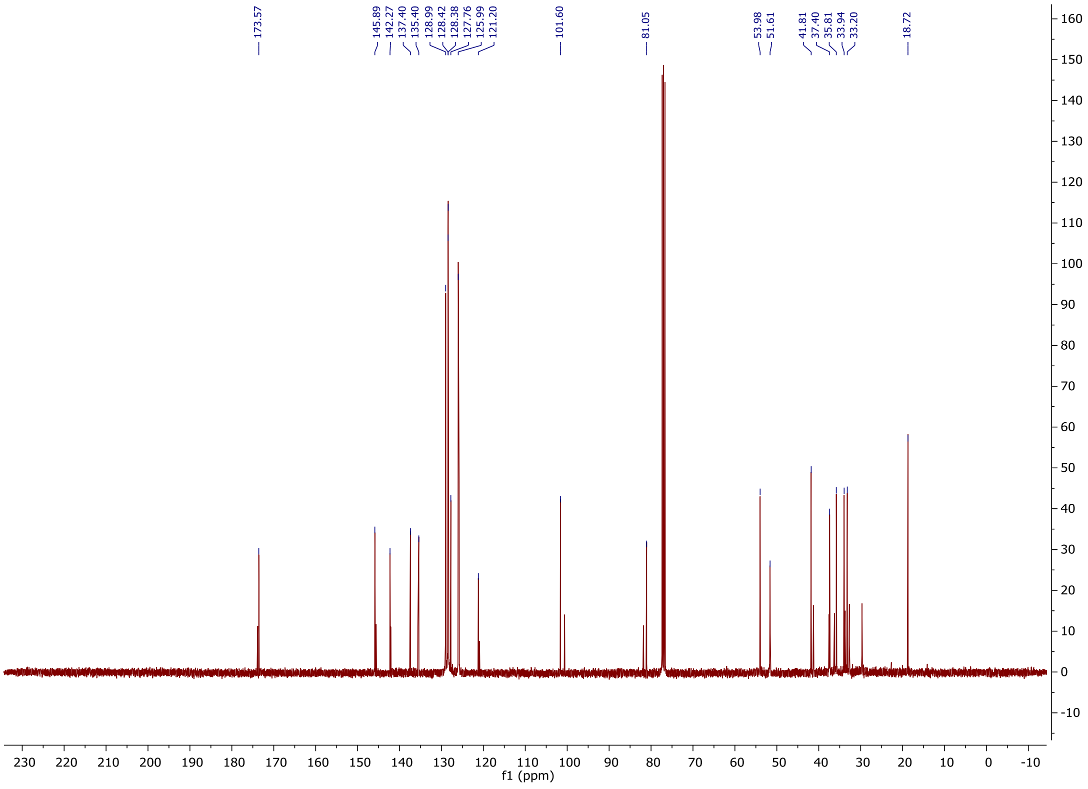
173.57
145.89 142.27 137.40 135.40 128.99 128.42 128.38 127.76 125.99 121.20
101.60
81.05
53.98 51.61
41.81 37.40 35.81 33.94 33.20
18.72
f1 (ppm)

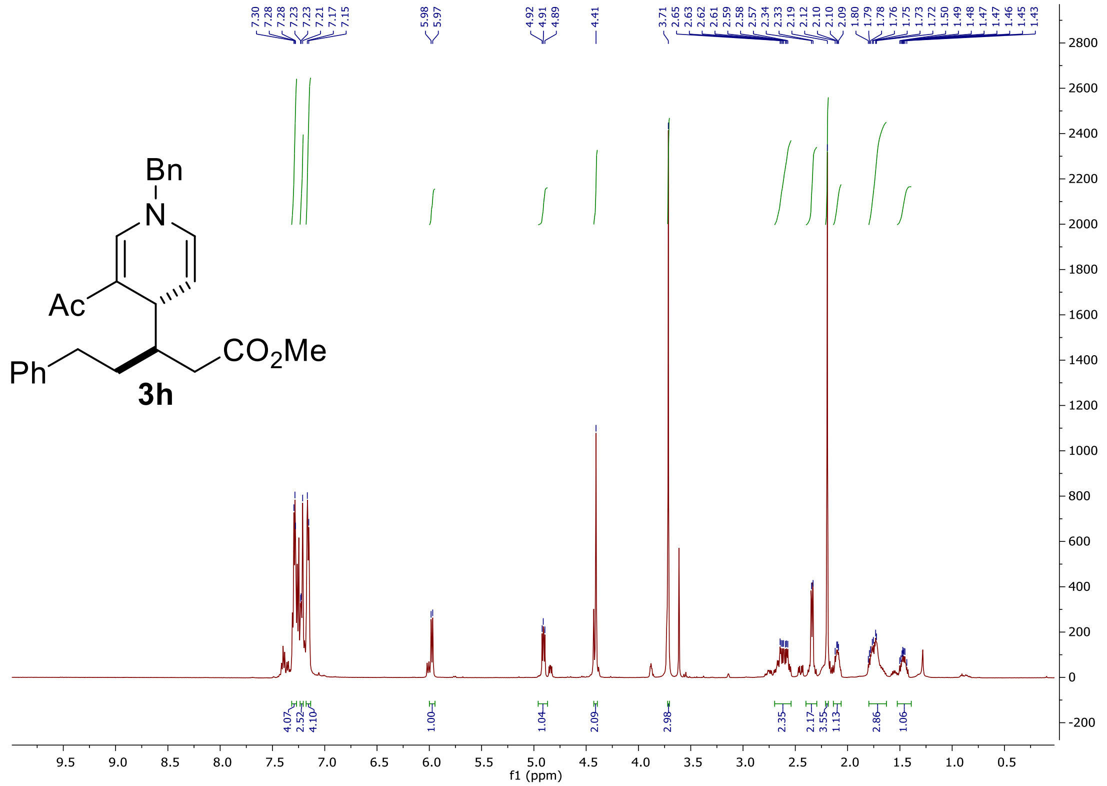
Bn
N
Ac
CO2Me
Ph
3h
f1 (ppm)

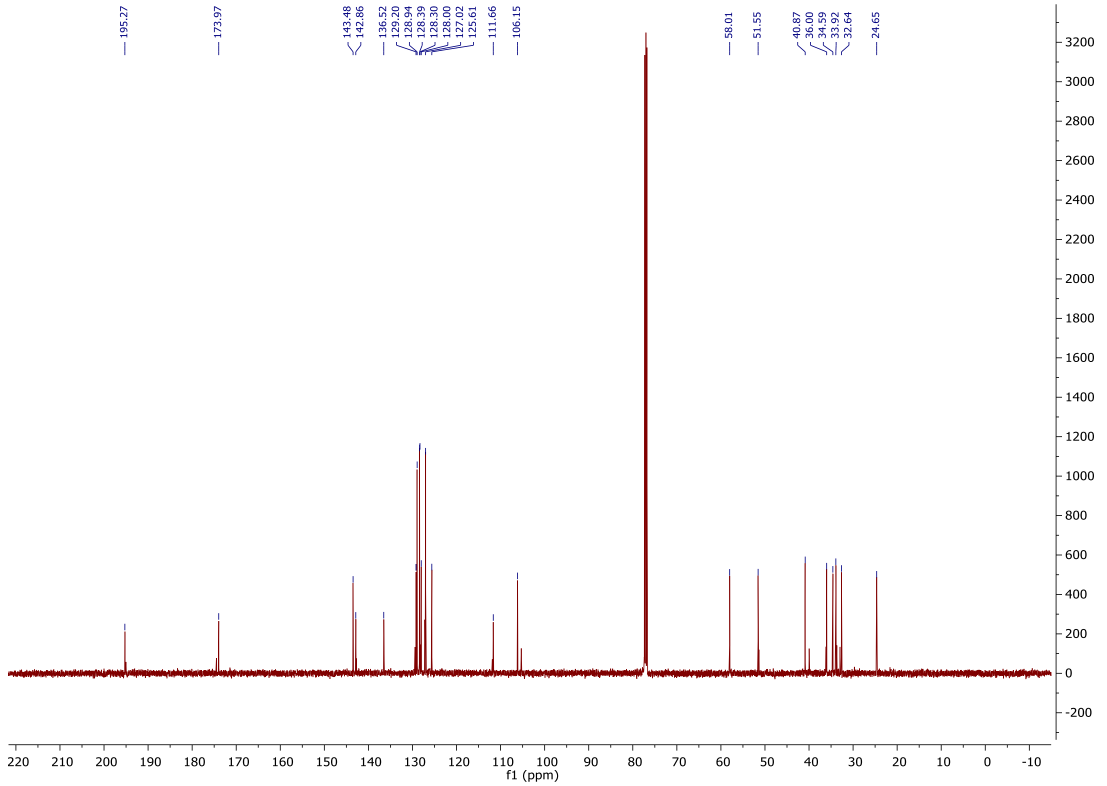
f1 (ppm)

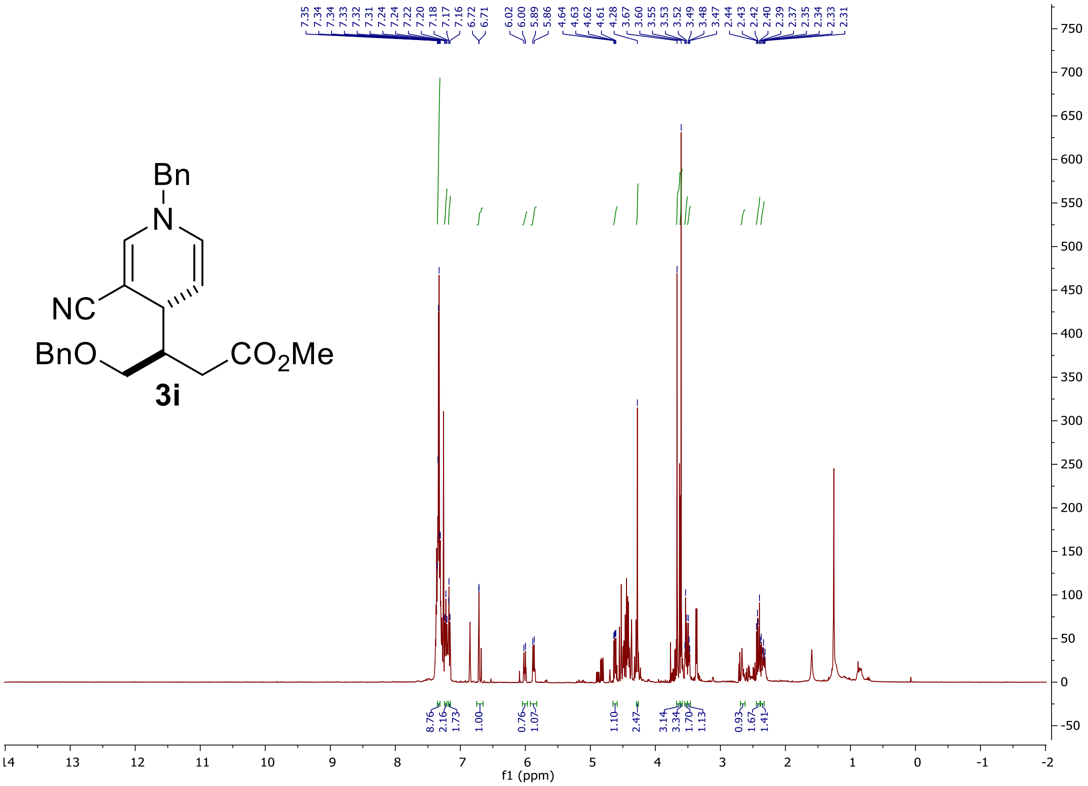
Bn
N
NC
BnO
CO2Me
3i
7.35 7.34 7.34 7.33 7.32 7.31 7.24 7.24 7.22 7.22 7.20 7.18 7.17 7.16 6.72 6.71 6.02 6.00 5.89 5.86 4.64 4.63 4.62 4.61 4.28 3.67 3.60 3.55 3.53 3.52 3.49 3.48 3.47 2.44 2.43 2.42 2.40 2.39 2.37 2.35 2.34 2.33 2.31
8.76 2.16 1.73 1.00 0.76 1.07 1.10 2.47 3.14 3.34 1.70 1.13 0.93 1.67 1.41
f1 (ppm)

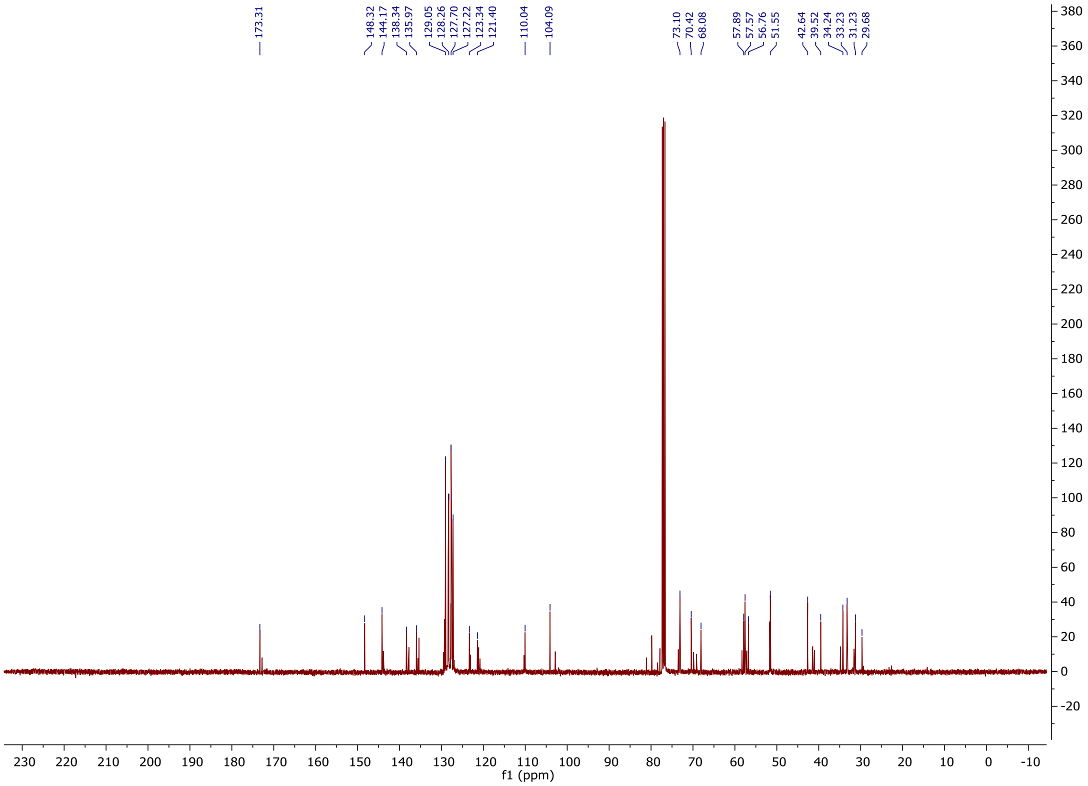
173.31
148.32 144.17 138.34 135.97 129.05 128.26 127.70 127.22 123.34 121.40
110.04 104.09
73.10 70.42 68.08 57.89 57.57 56.76 51.55 42.64 39.52 34.24 33.23 31.23 29.68
f1 (ppm)

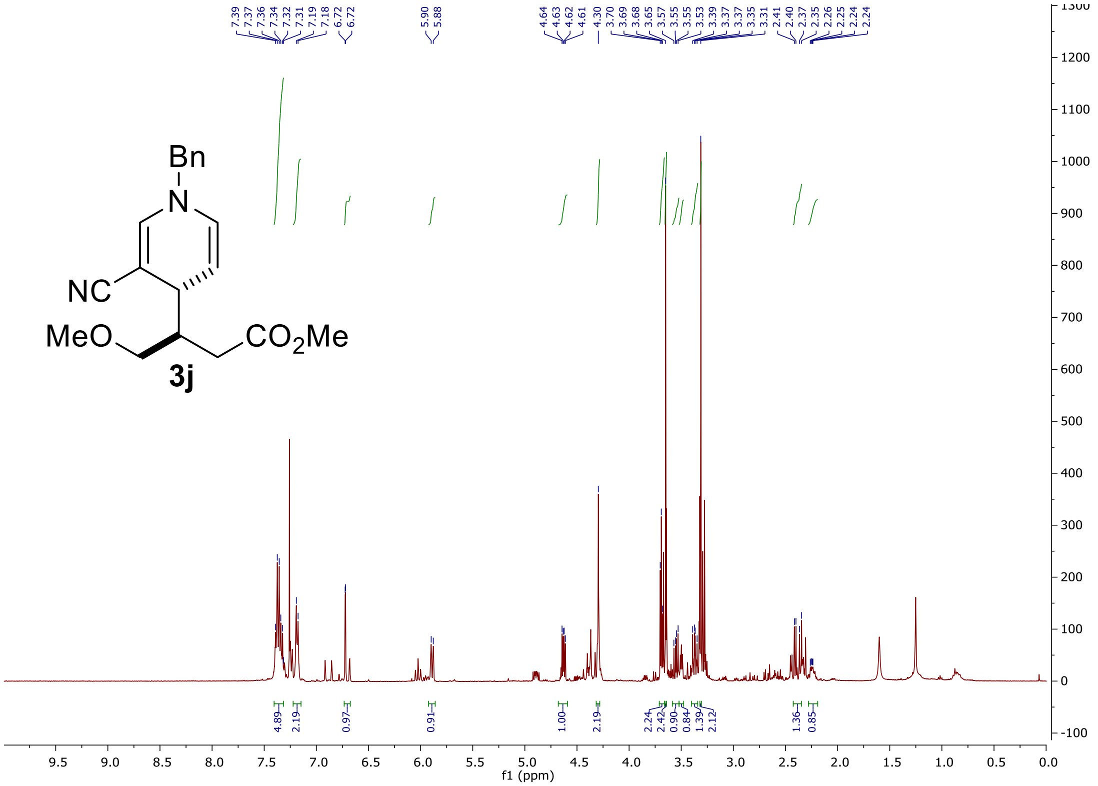
7.39 7.37 7.36 7.34 7.32 7.31 7.19 7.18 6.72 6.72
5.90 5.88
4.64 4.63 4.62 4.61 4.30 3.70 3.69 3.68 3.65 3.57 3.55 3.55 3.53 3.39 3.37 3.37 3.35 3.31 2.41 2.40 2.37 2.35 2.26 2.25 2.24 2.24
Bn
N
NC
MeO
CO2Me
3j
4.89 2.19 0.97 0.91 1.00 2.19 2.24 2.42 0.90 0.84 1.39 2.12 1.36 0.85
f1 (ppm)

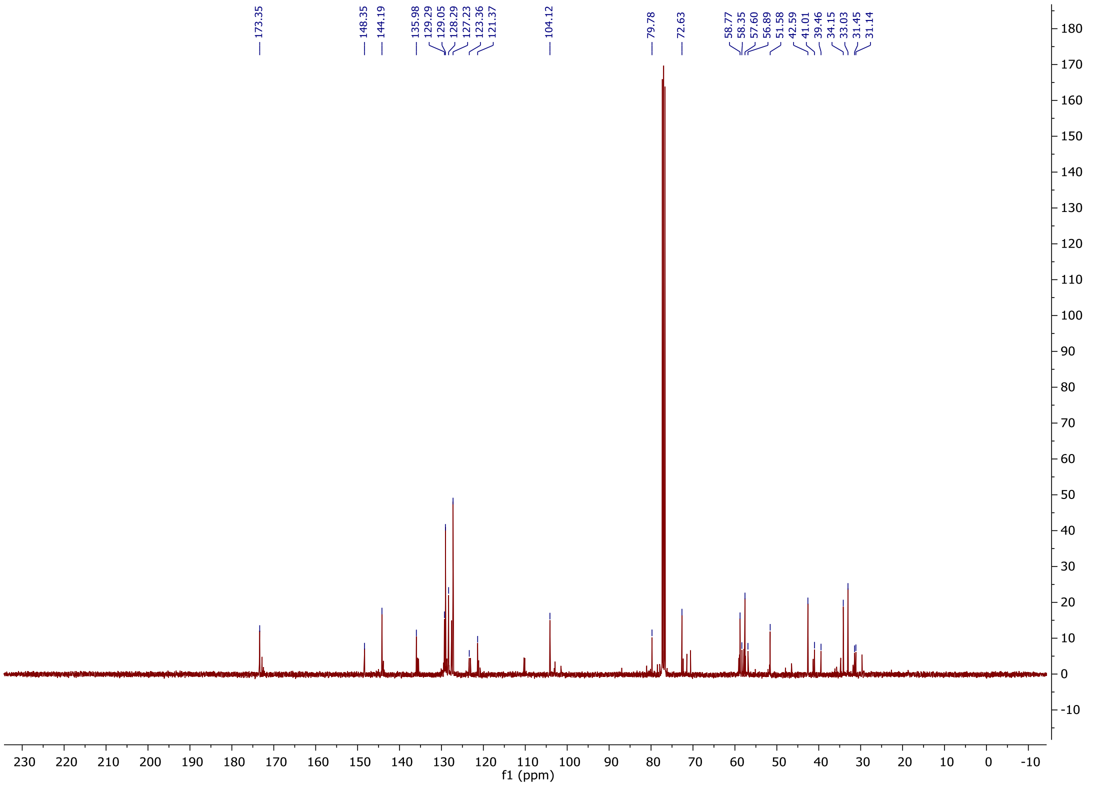
173.35
148.35 144.19
135.98 129.29 129.05 128.29 127.23 123.36 121.37
104.12
79.78
72.63
58.77 58.35 57.60 56.89 51.58 42.59 41.01 39.46 34.15 33.03 31.45 31.14
f1 (ppm)

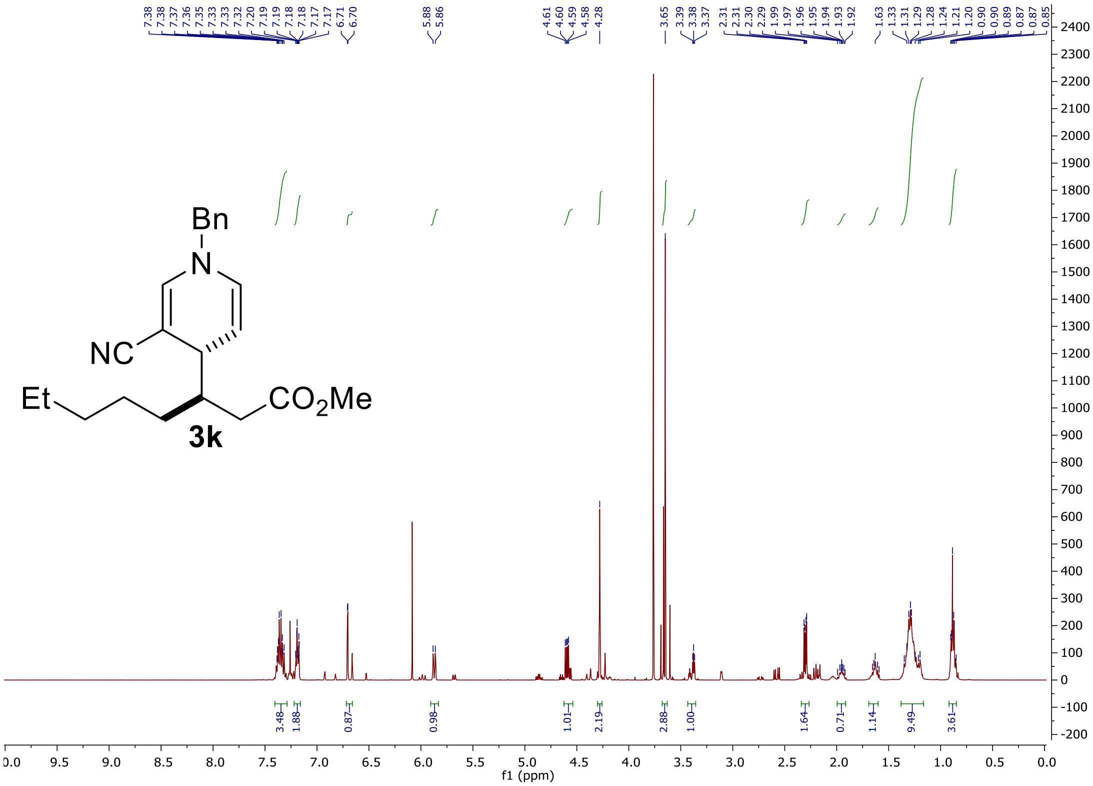
7.38
7.38
7.37
7.36
7.35
7.33
7.33
7.32
7.20
7.19
7.19
7.18
7.18
7.17
7.17
6.71
6.70
5.88
5.86
4.61
4.60
4.59
4.58
4.28
3.65
3.39
3.38
3.37
2.31
2.31
2.30
2.29
1.99
1.97
1.96
1.95
1.94
1.93
1.92
1.63
1.33
1.31
1.29
1.28
1.24
1.21
1.20
0.90
0.90
0.89
0.87
0.85
Bn
N
NC
Et
CO_2Me
3k
3.48
1.88
0.87
0.98
1.01
2.19
2.88
1.00
1.64
0.71
1.14
9.49
3.61
f1 (ppm)

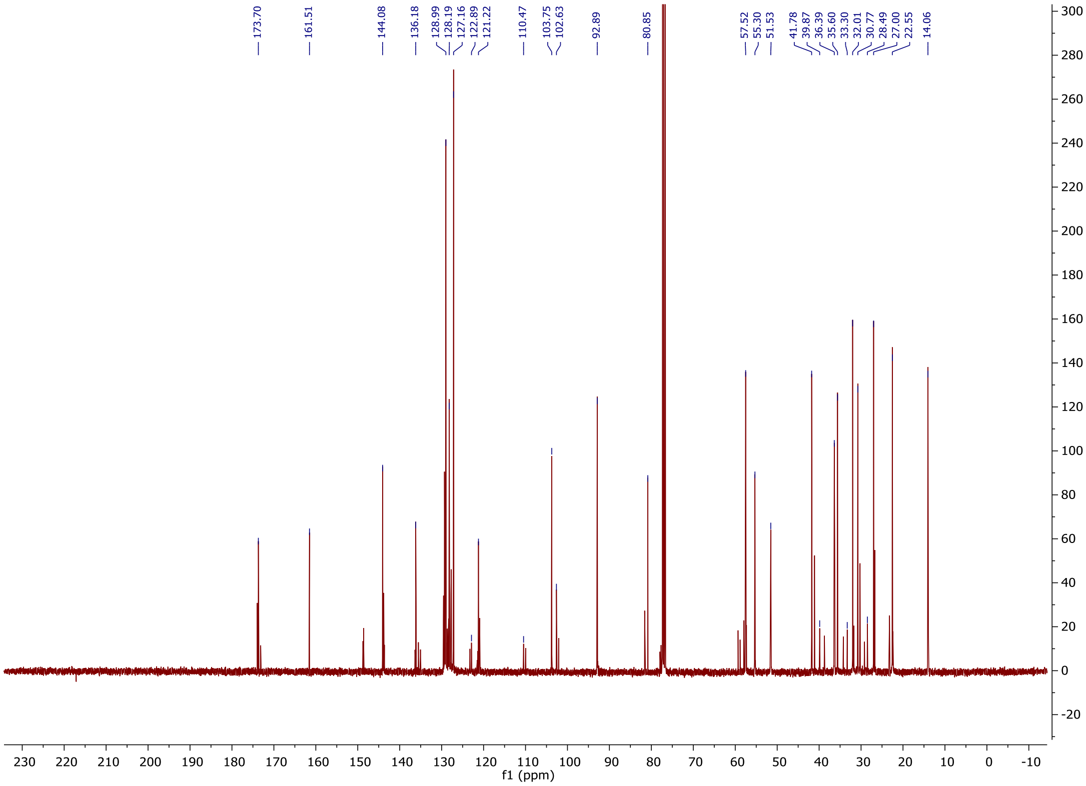
173.70
161.51
144.08
136.18
128.99
128.19
127.16
122.89
121.22
110.47
103.75
102.63
92.89
80.85
57.52
55.30
51.53
41.78
39.87
36.39
35.60
33.30
32.01
30.77
28.49
27.00
22.55
14.06
f1 (ppm)

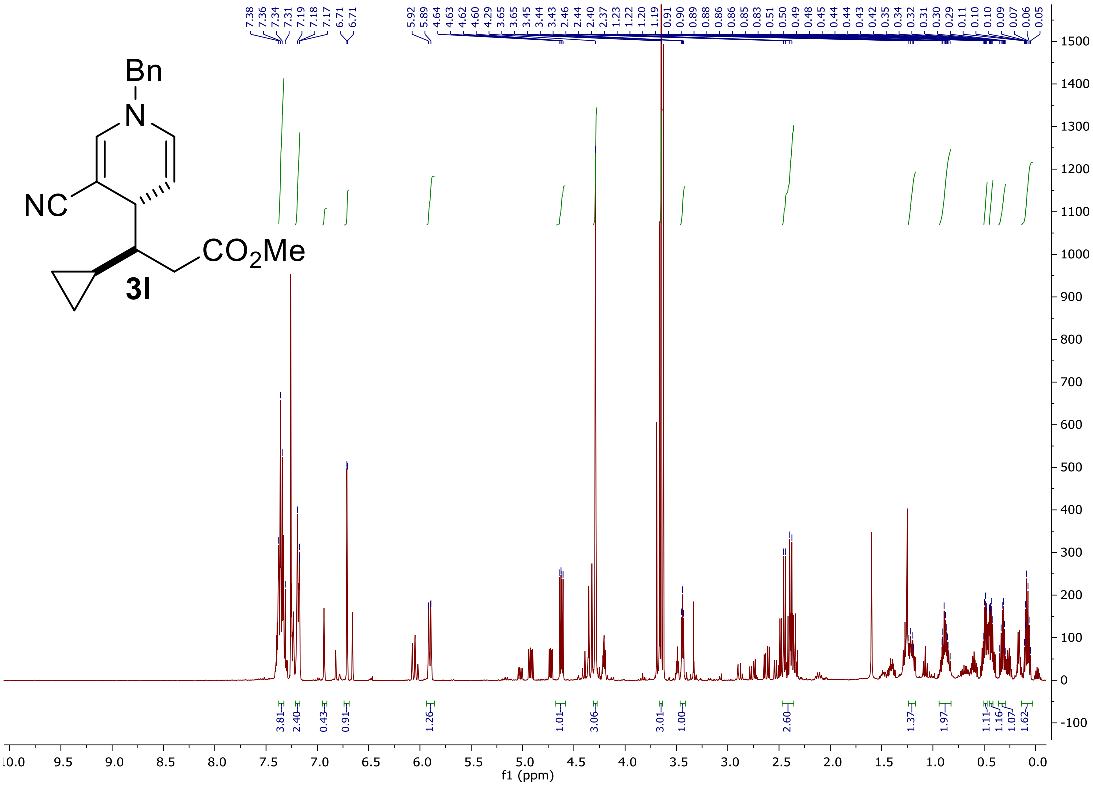

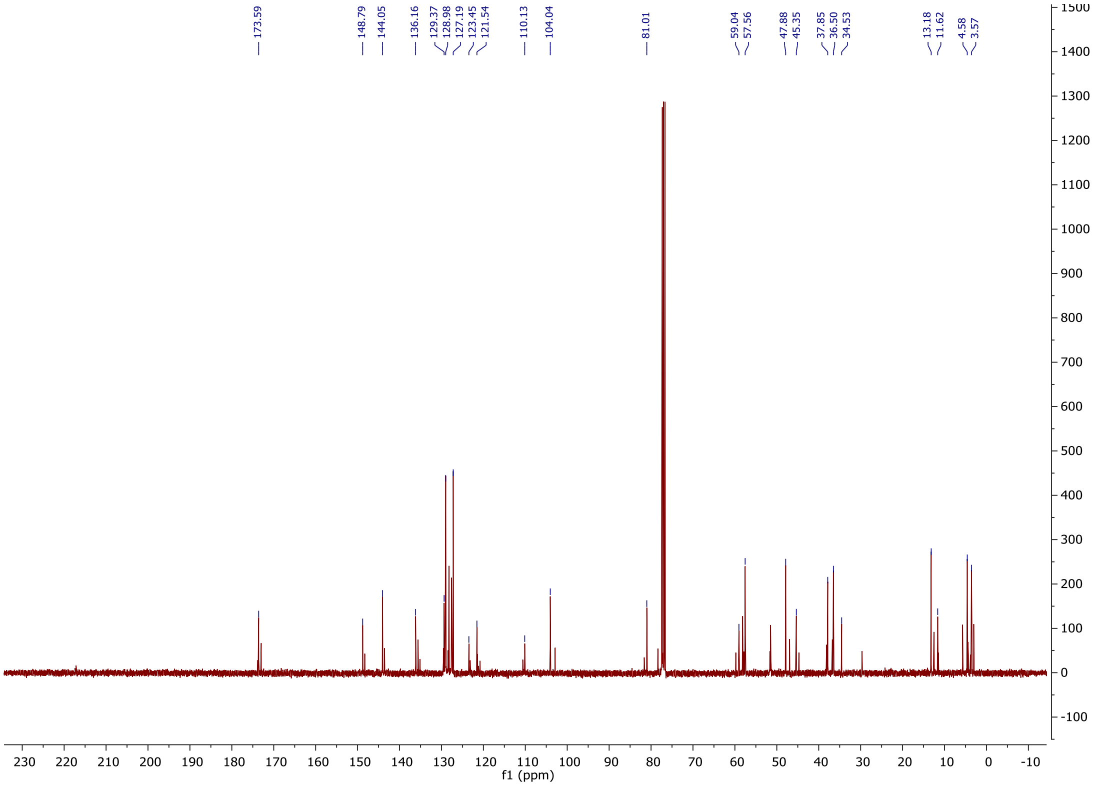

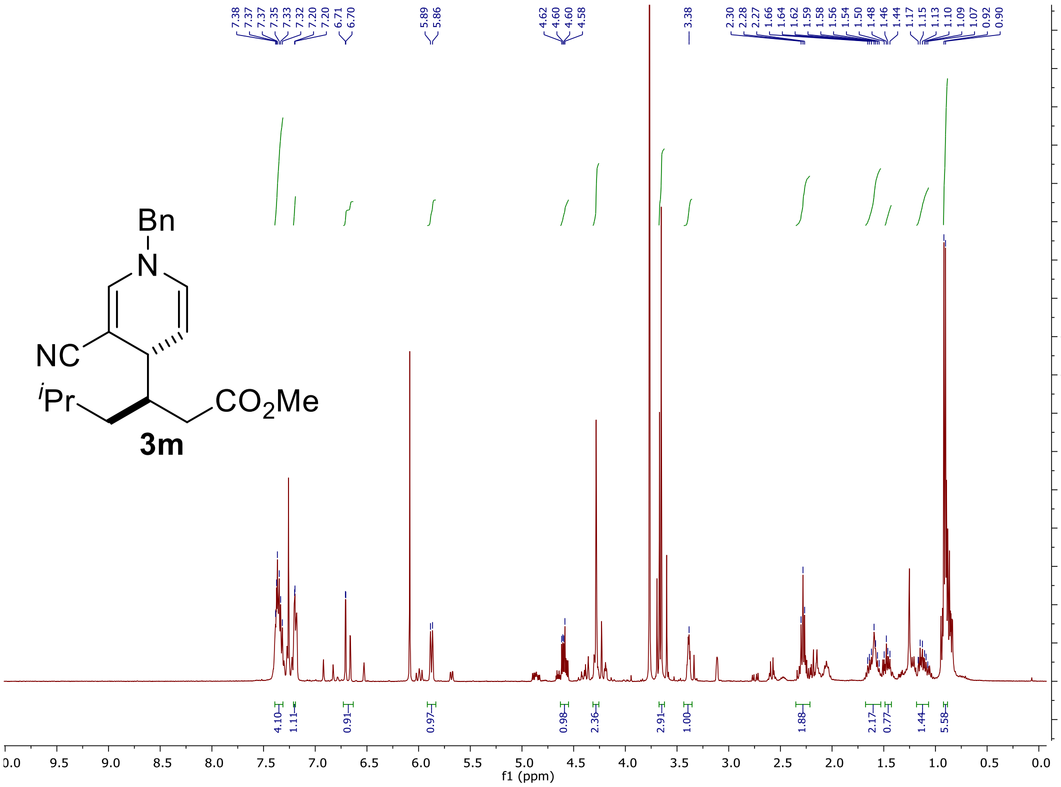
Bn
N
NC
iPr
CO2Me
3m
f1 (ppm)

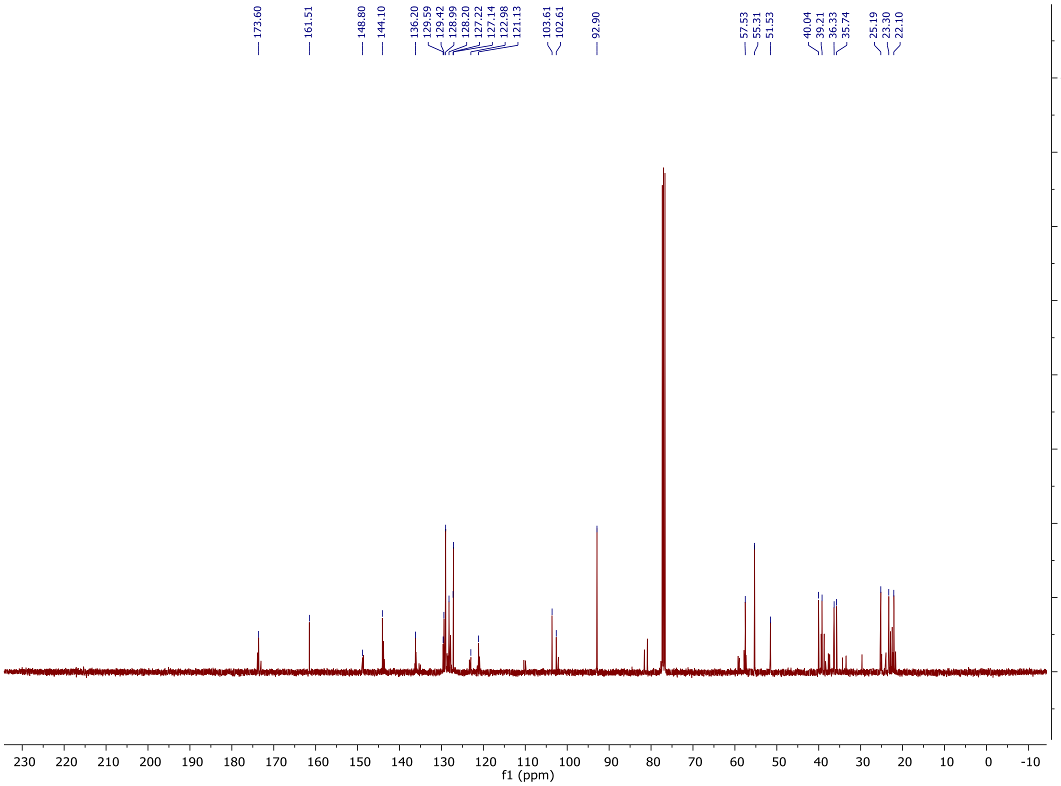
f1 (ppm)

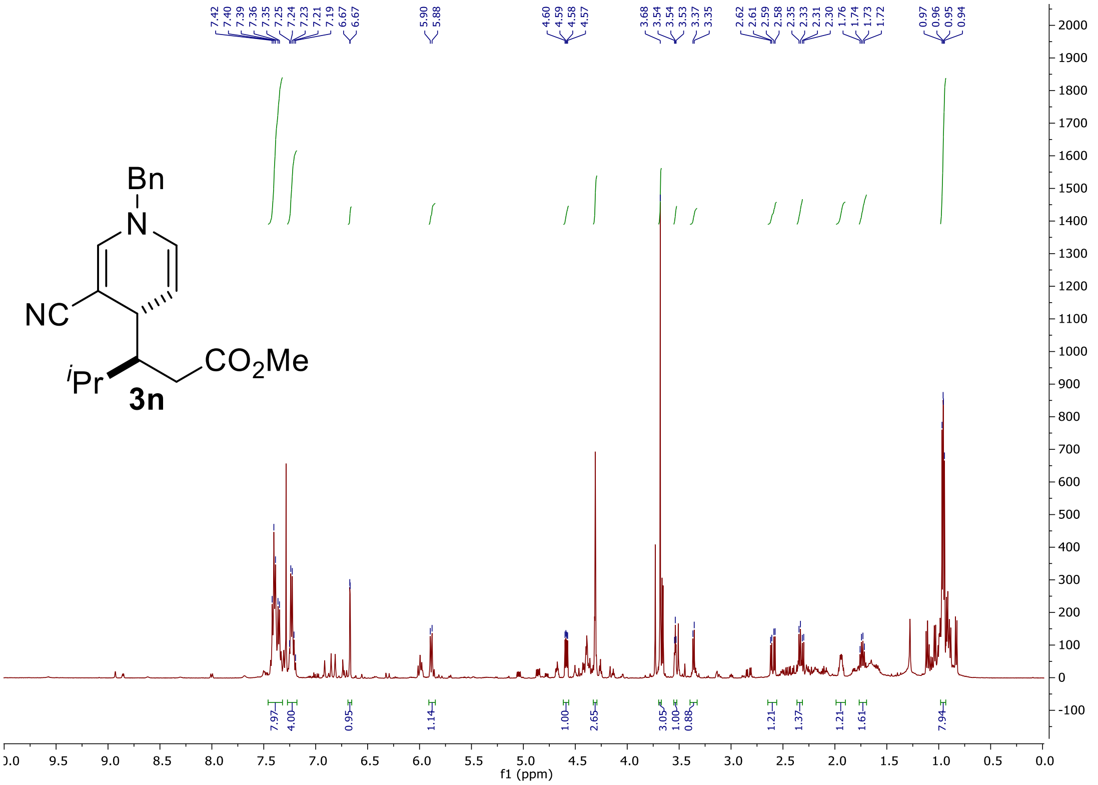
Bn
N
NC
CO2Me
iPr
7.42 7.40 7.39 7.36 7.35 7.25 7.24 7.23 7.21 7.19 6.67 6.67
5.90 5.88
4.60 4.59 4.58 4.57
3.68 3.54 3.54 3.53 3.37 3.35
2.62 2.61 2.59 2.58 2.35 2.33 2.31 2.30 1.76 1.74 1.73 1.72
0.97 0.96 0.95 0.94
7.97 4.00 0.95 1.14 1.00 2.65 3.05 1.00 0.88 1.21 1.37 1.21 1.61 7.94
f1 (ppm)

3n

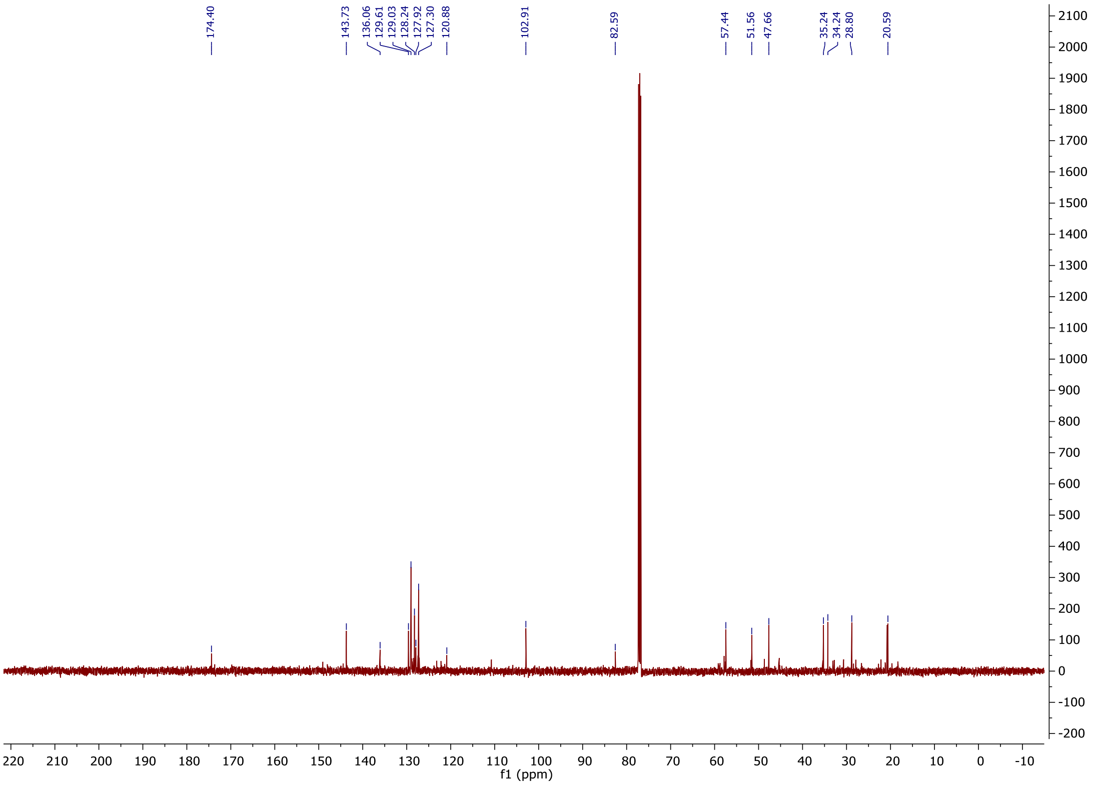
174.40
143.73 136.06 129.61 129.03 128.24 127.92 127.30 120.88
102.91
82.59
57.44 51.56 47.66
35.24 34.24 28.80
20.59
f1 (ppm)

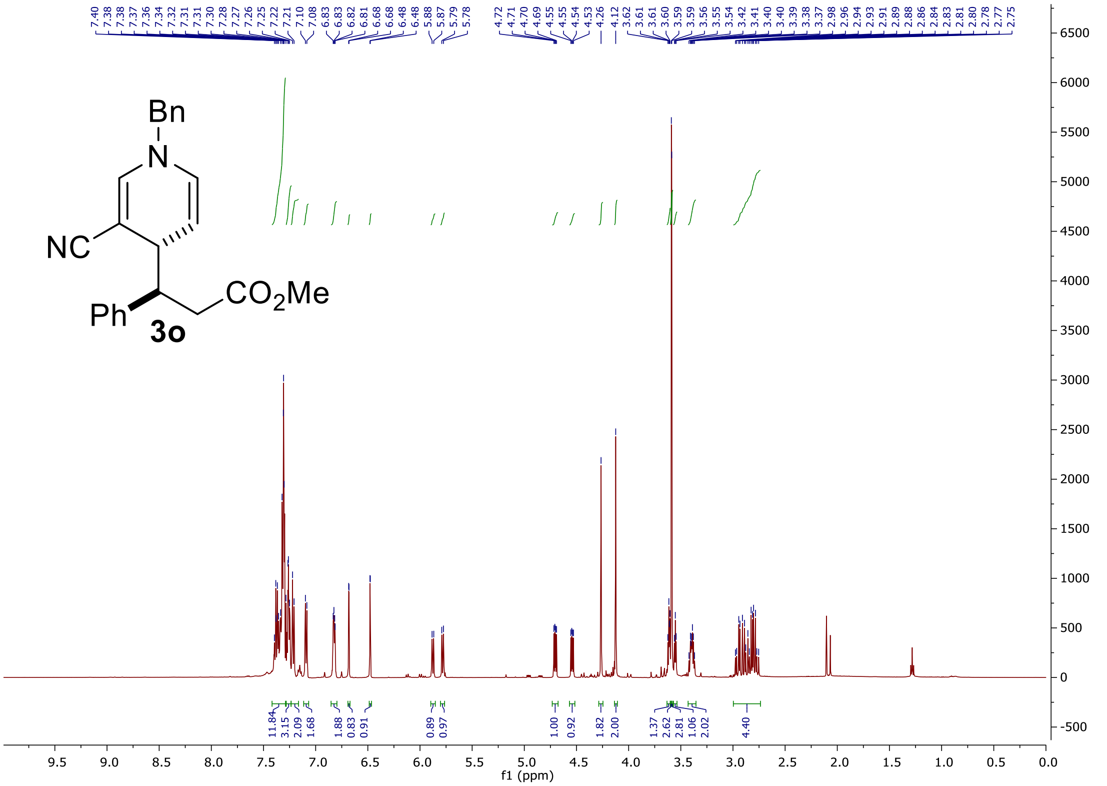

7.40 7.38 7.38 7.37 7.36 7.34 7.32 7.31 7.31 7.30 7.28 7.27 7.26 7.25 7.22 7.21 7.10 7.08 6.83 6.83 6.82 6.81 6.68 6.68 6.48 6.48 5.88 5.87 5.79 5.78
4.72 4.71 4.70 4.69 4.55 4.55 4.54 4.53 4.26 4.12 3.62 3.61 3.61 3.60 3.59 3.59 3.56 3.55 3.54 3.42 3.41 3.40 3.40 3.39 3.38 3.37 2.98 2.96 2.94 2.93 2.91 2.89 2.88 2.86 2.84 2.83 2.81 2.80 2.78 2.77 2.75
Bn
N
NC
CO2Me
Ph
3o
11.84 3.15 2.09 1.68 1.88 0.83 0.91 0.89 0.97 1.00 0.92 1.82 2.00 1.37 2.62 2.81 1.06 2.02 4.40
9.5 9.0 8.5 8.0 7.5 7.0 6.5 6.0 5.5 5.0 4.5 4.0 3.5 3.0 2.5 2.0 1.5 1.0 0.5 0.0
f1 (ppm)
6500 6000 5500 5000 4500 4000 3500 3000 2500 2000 1500 1000 500 0 -500

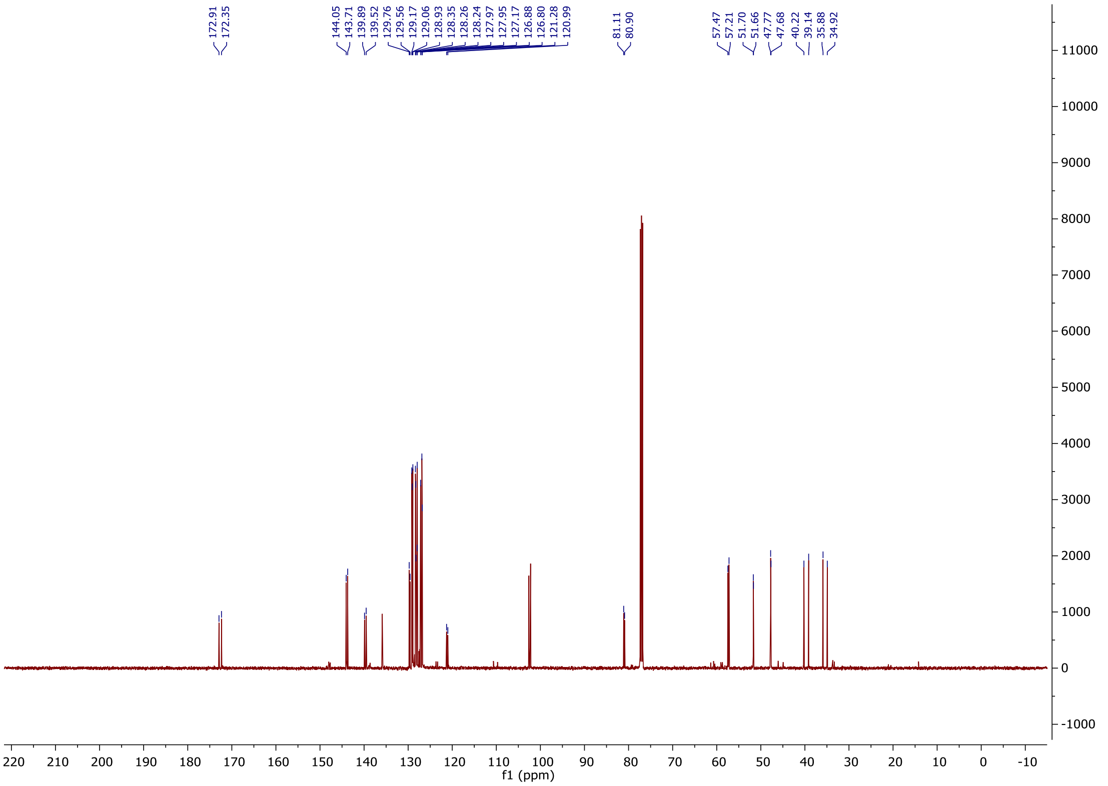

172.91 172.35
144.05 143.71 139.89 139.52 129.76 129.56 129.17 129.06 128.93 128.35 128.26 128.24 127.97 127.95 127.17 126.88 126.80 121.28 120.99
81.11 80.90
57.47 57.21 51.70 51.66 47.77 47.68 40.22 39.14 35.88 34.92
220 210 200 190 180 170 160 150 140 130 120 110 100 90 80 70 60 50 40 30 20 10 0 -10
f1 (ppm)
11000 10000 9000 8000 7000 6000 5000 4000 3000 2000 1000 0 -1000

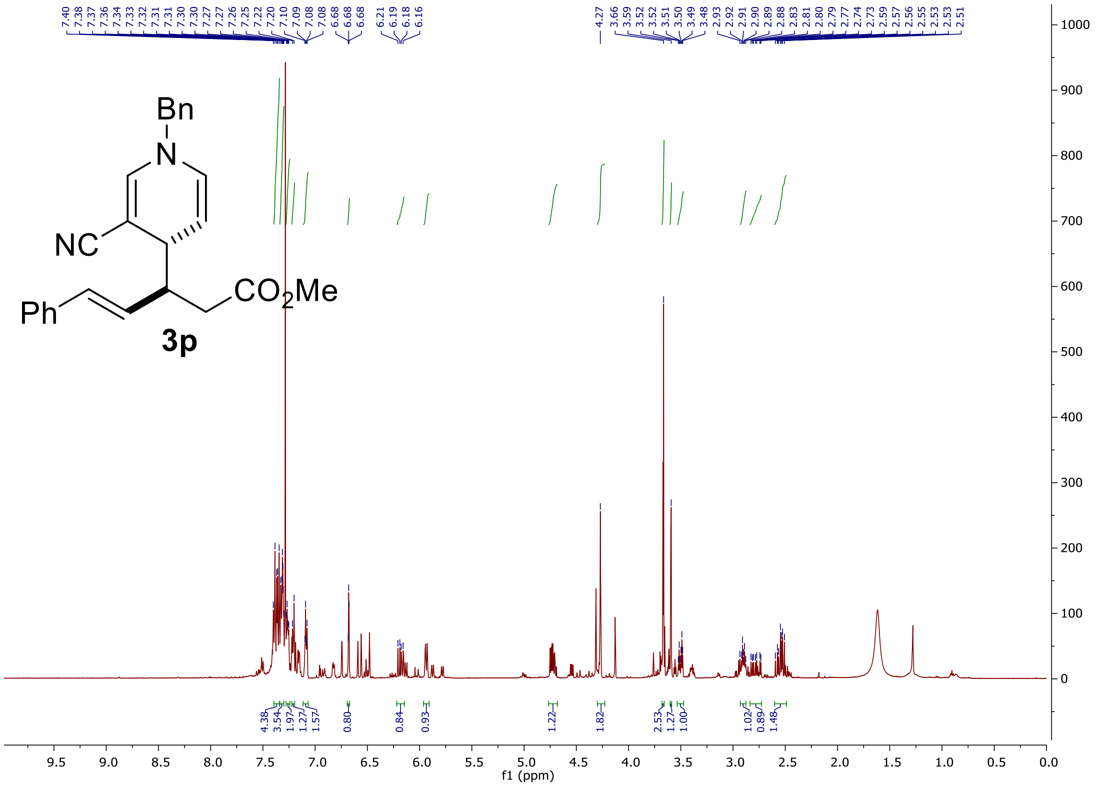
7.40 7.38 7.37 7.36 7.34 7.33 7.32 7.31 7.31 7.30 7.30 7.27 7.27 7.26 7.25 7.22 7.20 7.10 7.09 7.08 7.08 6.68 6.68 6.68 6.21 6.19 6.18 6.16
4.27 3.66 3.59 3.52 3.52 3.51 3.50 3.49 3.48 2.93 2.92 2.91 2.90 2.89 2.88 2.83 2.81 2.80 2.79 2.77 2.74 2.73 2.59 2.57 2.56 2.55 2.53 2.53 2.51
Bn
N
NC
Ph
CO_2Me
3p
4.38 3.54 1.97 1.27 1.57 0.80 0.84 0.93 1.22 1.82 2.53 1.27 1.00 1.02 0.89 1.48
f1 (ppm)

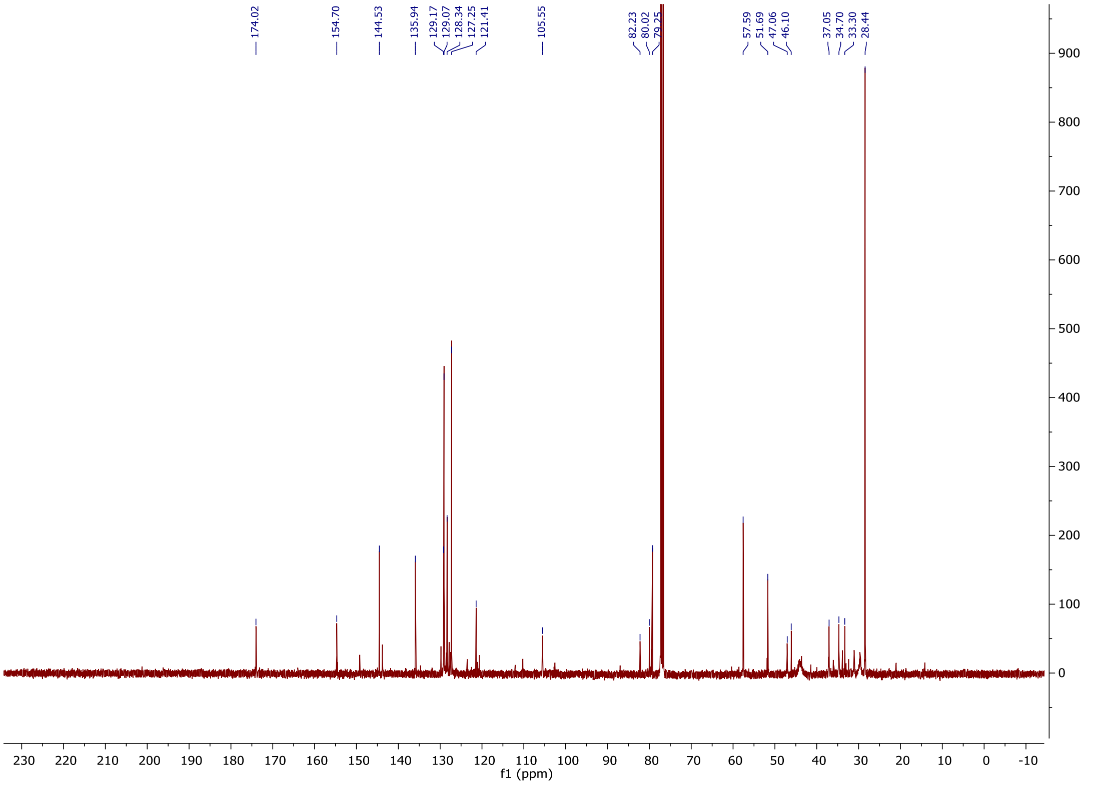
174.02 154.70 144.53 135.94 129.17 129.07 128.34 127.25 121.41 105.55 82.23 80.02 79.25 57.59 51.69 47.06 46.10 37.05 34.70 33.30 28.44
f1 (ppm)

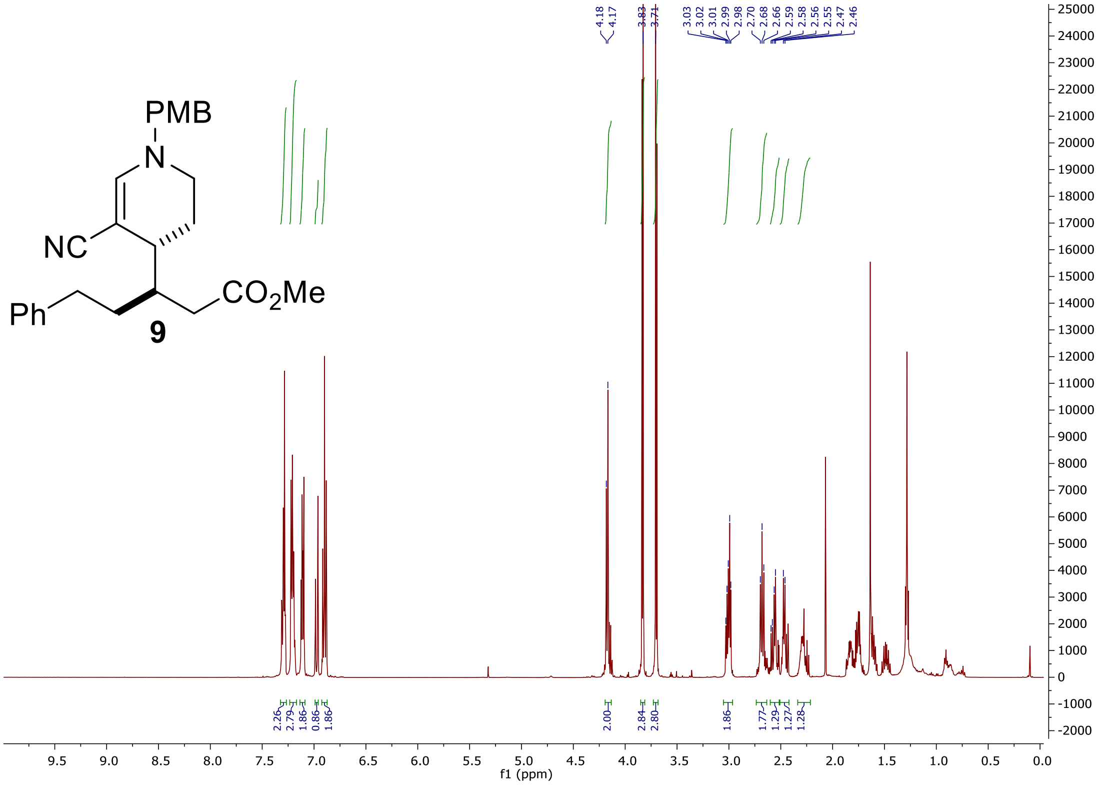
PMB
N
NC
CO2Me
Ph
9
4.18
4.17
3.83
3.71
3.03
3.02
3.01
2.99
2.98
2.70
2.68
2.66
2.59
2.58
2.56
2.55
2.47
2.46
2.26
2.79
1.86
0.86
1.86
2.00
2.84
2.80
1.86
1.77
1.29
1.27
1.28
f1 (ppm)

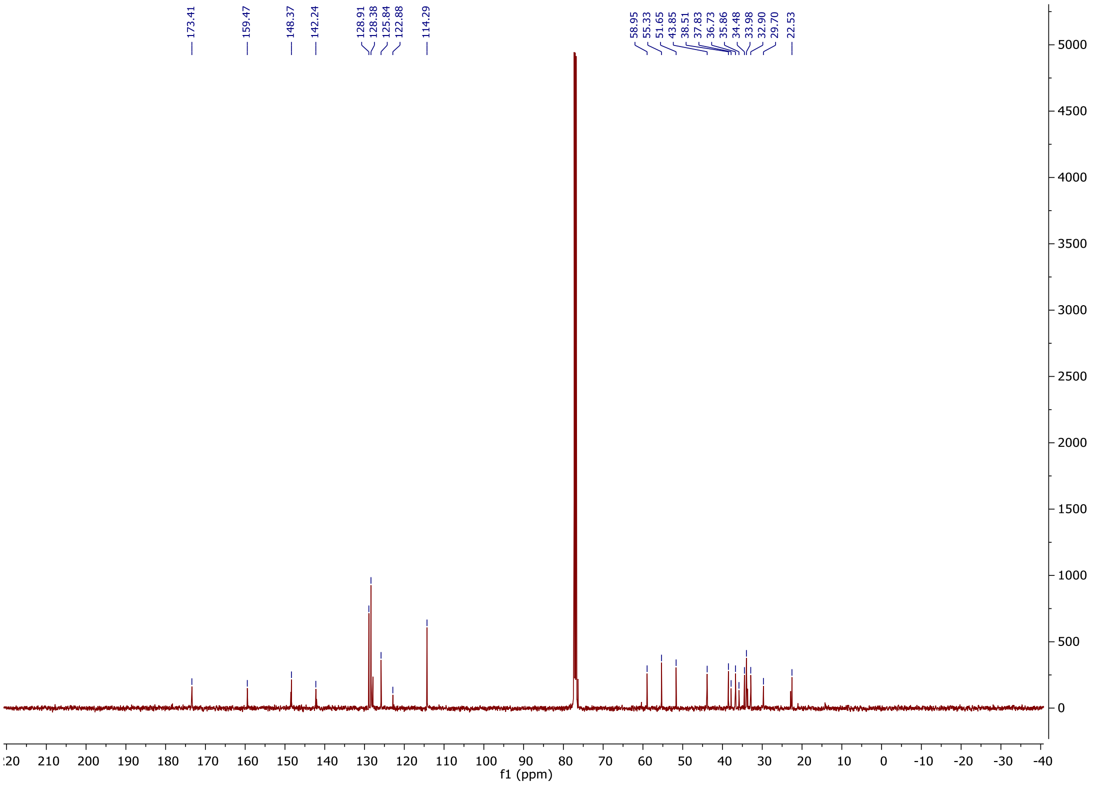
173.41
159.47
148.37
142.24
128.91
128.38
125.84
122.88
114.29
58.95
55.33
51.65
43.85
38.51
37.83
36.73
35.86
34.48
33.98
32.90
29.70
22.53
f1 (ppm)

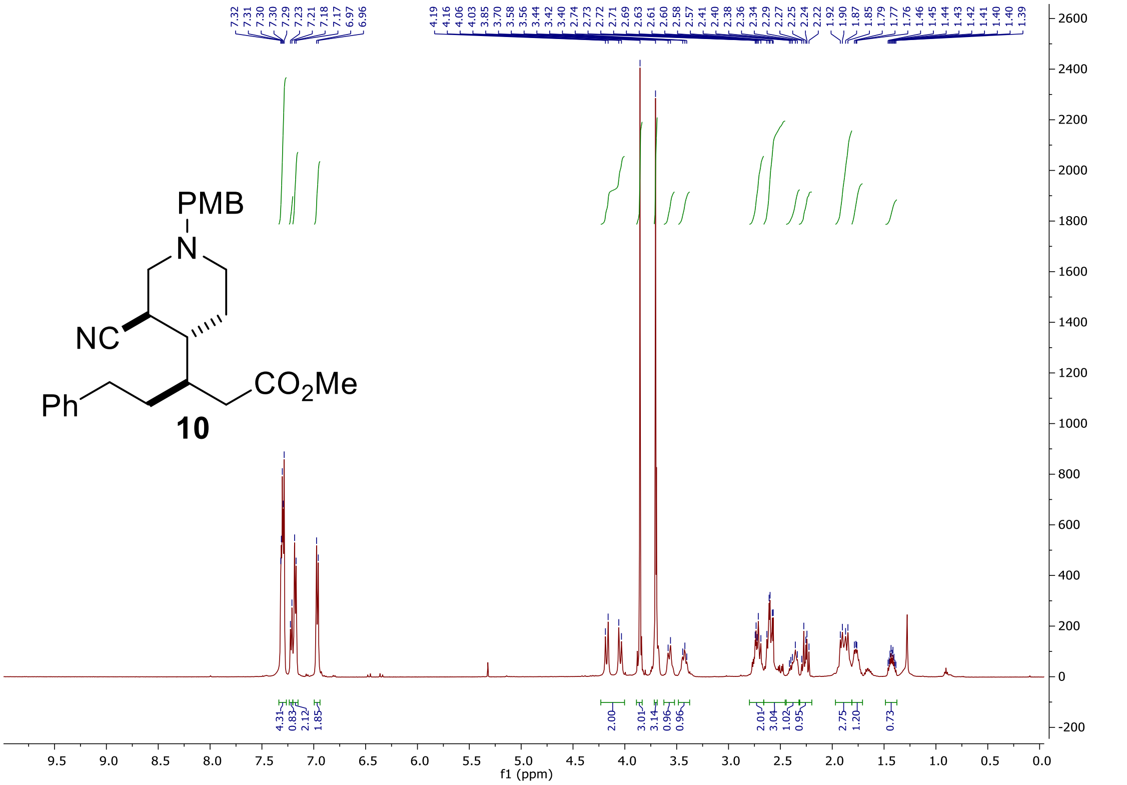

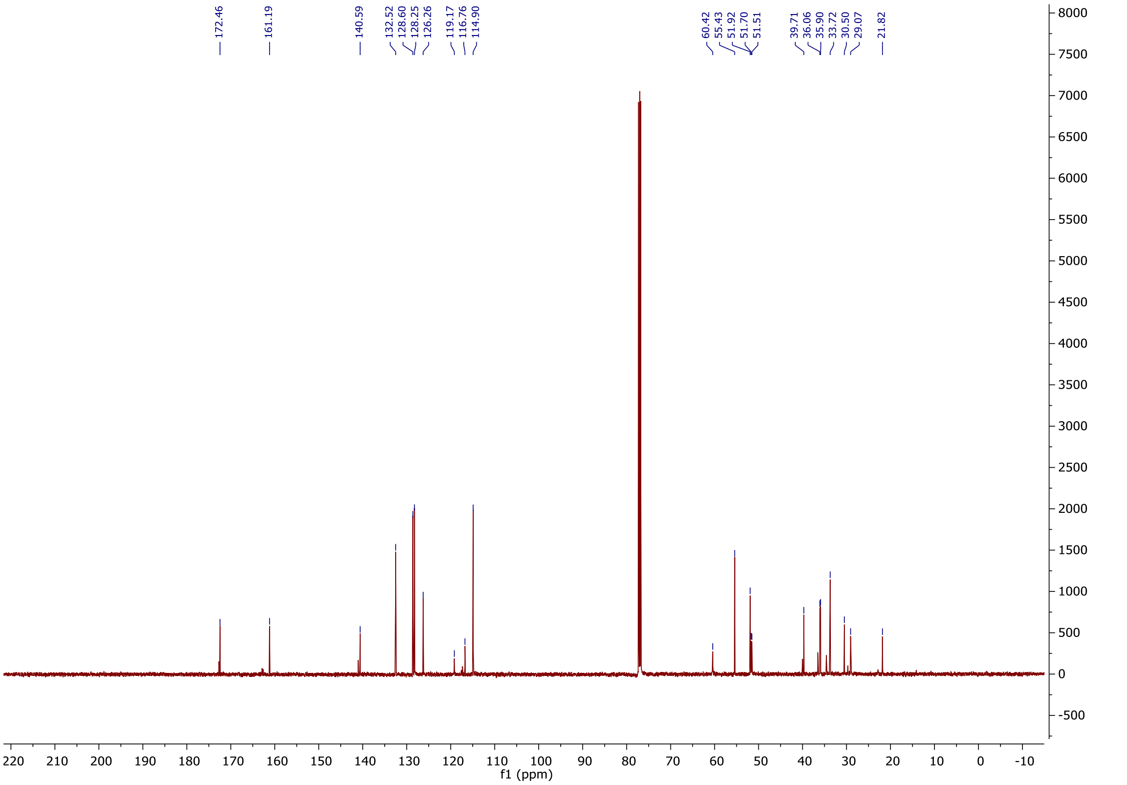

Single crystal X-ray diffraction for compound 9.

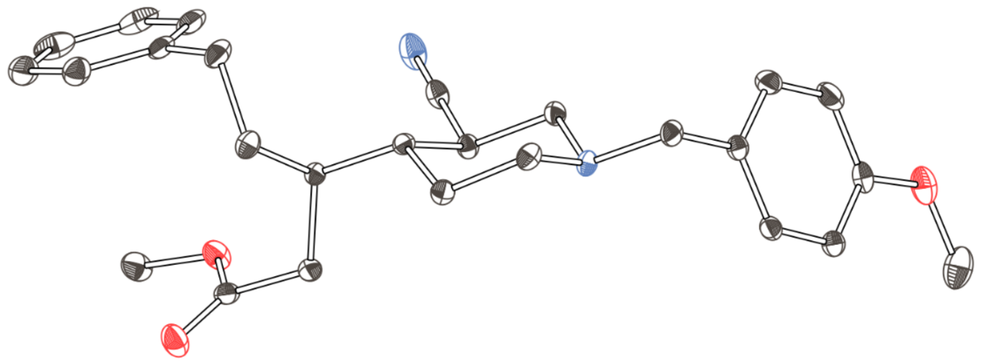

Single crystal X-ray diffraction.[1] Data for all compounds was collected on an Agilent SuperNova diffractometer using mirror-monochromated Cu Kα. Data collection, integration, scaling (ABSPACK) and absorption correction (face-indexed Gaussian integration2 or numeric analytical methods3) were performed in CrysAlisPro.4 Structure solution was performed using ShelXT.[5] Subsequent refinement was performed by full-matrix least-squares on F^2 in ShelXL. Olex26 was used for viewing and to prepare CIF files. PLATON7 was used for Bijvoet difference analysis of absolute structure (further details within). ORTEP graphics were prepared in CrystalMaker.8 Thermal ellipsoids are rendered at the 50% probability level.

A single crystal of **9** was grown from a sample of 94% enantiomeric purity as determined by chiral HPLC. A dichloromethan solution was diluted with pentane by vapor diffusion to afford apque, colorless blades. Part of a crystal (.20 x .05 x .05 mm) was separated carefully, mounted with STP oil treatment, and cooled to 100 K on the diffractometer. A full sphere of data were collected to 0.800 Å resolution. 43306 reflections were collected (4462 unique, 4366 observed) with R(int) 4.2% and R(sigma) 2.0% after Gaussian absorption and beam profile correction (Tmin .910, Tmax .977).

The space group was assigned as $P2_12_12_1$ based on the systematic absences. The structure solved routinely in ShelXT with 1 molecule in the asymmetric unit. All non-H atoms were located in the initial solution and refined anisotropically with no restraints. C-H hydrogens were placed in calculated positions and refined with riding coordinates and ADPs.

---

1 Single crystal X-ray diffraction was performed at the Shared Materials Characterization Laboratory at Columbia University. Use of the SMCL was made possible by funding from Columbia University.
2 Blanc, E.; Schwarzenbach, D.; Flack, H. D. *J. Appl. Cryst.* **24** (1991), 1035-1041.
3 Clark. R. C.; Reid, J. S. *Acta Cryst.* **A51** (1995), 887-897.
4 Version 1.171.37.35 (2014). Oxford Diffraction /Agilent Technologies UK Ltd, Yarnton, England.
5 Sheldrick, G. M. *Acta Cryst.* **A71** (2015), 3-8.
6 Dolomanov, O. V.; Bourhis, L. J.; Gildea, R. J.; Howard, J. A. K.; Puschmann, H. *J. Appl. Cryst*. **42** (2009), 339-341.
7 Spek, A. *Acta Cryst*. **D65** (2009), 148-155.
8 CrystalMaker Software Ltd, Oxford, England *(www.crystalmaker.com)*.

The final refinement (462 data, 0 restraints, 282 parameters) converged with R_1 ($F_o > 4\sigma(F_o)$) = 2.9%, wR_2 = 7.4%, S = 1.05. The largest Fourier features were 0.16 and -0.15 $e^- A^{-3}$.

A crystal with molecular formula $C_{26}H_{32}N_2O_3$ is a reasonable target for absolute structure determination using Cu Kα radiation.9 The Flack x parameter was 0.07(6) by the Parsons selected quotients method implemented in ShelXL. For confirmation of the absolute structure, the data set was analyzed by the probabilistic approach of Hooft, Straver, and Spek10 as implemented in PLATON. Errors were assumed to be Gaussian; a normal probabilities plot was linear with correlation coefficient 0.999 and slope 0.956. Using an outlier criterion of 79.66 and sigma criterion of 0.25, 404 Bijvoet pairs were selected for analysis. The Hooft y parameter was 0.06(6) using these parameters. The probability of a racemic twin, P3(rac-twin), was calculated as 4 x 10^{-14} and the probability of an incorrect absolute structure, P3(false), was 4 x 10^{-63}. Therefore, we state with high confidence that the absolute structure is correctly assigned.

Figure Sx. Molecular structure of **9**.

**Compound**	**9**
**Formula**	$C_{26}H_{32}N_2O_3$
**MW**	420.53
**Space group**	$P2_12_12_1$
a **(Å)**	6.27180(10)
b **(Å)**	14.71860(10)
c **(Å)**	24.2813(2)
α **(°)**	90
β **(°)**	90
γ **(°)**	90
**V (Å^3)**	2241.46(4)
**Z**	4

[9] Parsons, S.; Flack, H. D.; Wagner, T. *Acta Cryst.* **B69** (2013), 249-259.
10 Hooft, R. W. W.; Straver, L. H.; Spek, A. L. *J. Appl. Cryst.* **41** (2008), 96-103.

**ρ_{calc} (g cm^{-3})**	1.246
**T (K)**	100
**λ (Å)**	1.54184
**$2\theta_{min}$, $2\theta_{max}$**	7, 146
**Nref**	43306
**R(int), R(σ)**	.0424, .0200
**μ(mm^{-1})**	0.645
**Size (mm)**	.20 x .05 x .05
**T_{max}, T_{min}**	.977, .910
**Data**	4462
**Restraints**	0
**Parameters**	282
**R_1(obs)**	0.0288
**wR_2(all)**	0.0743
**S**	1.045
**Peak, hole (e$^-$ Å^{-3})**	0.16, -0.15
**Hooft y**	0.06(6)

Table 1 Crystal data and structure refinement for bcorr_a.

Identification code	bcorr_a
Empirical formula	$C_{26}H_{32}N_2O_3$
Formula weight	420.53
Temperature/K	100.01(10)
Crystal system	orthorhombic
Space group	$P2_12_12_1$
a/Å	6.27180(10)
b/Å	14.71860(10)
c/Å	24.2813(2)
α/°	90
β/°	90

γ/°	90
Volume/Å^3	2241.46(4)
Z	4
ρ_{calc}g/cm^3	1.246
μ/mm^{-1}	0.645
F(000)	904.0
Crystal size/mm^3	0.196 × 0.049 × 0.048
Radiation	CuKα (λ = 1.54184)
2Θ range for data collection/°	7.022 to 145.98
Index ranges	-7 ≤ h ≤ 7, -18 ≤ k ≤ 18, -29 ≤ l ≤ 30
Reflections collected	43306
Independent reflections	4462 [R_{int} = 0.0424, R_{sigma} = 0.0200]
Data/restraints/parameters	4462/0/282
Goodness-of-fit on F^2	1.045
Final R indexes [I>=2σ (I)]	R_1 = 0.0288, wR_2 = 0.0737
Final R indexes [all data]	R_1 = 0.0296, wR_2 = 0.0743
Largest diff. peak/hole / e Å^{-3}	0.16/-0.15
Flack parameter	0.07(6)

Table 2 Fractional Atomic Coordinates (×10^4) and Equivalent Isotropic Displacement Parameters (Å^2×10^3) for bcorr_a. U_{eq} is defined as 1/3 of of the trace of the orthogonalised U_{IJ} tensor.

**Atom**	***x***	***y***	***z***	**U(eq)**
N1	119(2)	6276.2(9)	4025.4(5)	14.3(3)
C2	1459(3)	5863.1(10)	3599.9(6)	14.5(3)
C3	1802(3)	4848(1)	3725.0(6)	13.8(3)
C4	-340(3)	4333.5(10)	3765.3(6)	12.8(3)
C5	-1718(3)	4828.5(11)	4189.4(7)	15.3(3)
C6	-1977(3)	5828.5(11)	4041.4(7)	16.0(3)
C7	-160(3)	7255.8(11)	3914.0(7)	16.4(3)
C8	1881(3)	7787.7(10)	3960.5(7)	15.4(3)
C9	2978(3)	8097.8(11)	3497.2(7)	19.8(3)
C10	4843(3)	8597.6(12)	3544.5(7)	21.7(3)
C11	5639(3)	8806.5(10)	4066.3(7)	17.7(3)
C12	4574(3)	8509.4(11)	4535.4(7)	16.5(3)
C13	2721(3)	7999.8(10)	4476.1(6)	15.2(3)

O14	7478(2)	9307.6(9)	4077.0(6)	26.6(3)
C15	8326(3)	9528.5(14)	4604.8(9)	31.0(4)
C16	3128(3)	4455.1(11)	3285.4(7)	16.9(3)
N17	4098(3)	4146.8(10)	2931.3(7)	24.8(3)
C18	-48(3)	3308.4(10)	3871.2(6)	12.9(3)
C19	699(3)	3095.3(11)	4462.0(6)	15.1(3)
C20	1311(3)	2110.6(11)	4515.1(6)	15.5(3)
O21	509(2)	1566.1(9)	4822.5(5)	24.8(3)
O22	2912(2)	1902.1(8)	4168.6(5)	20.4(3)
C23	3544(3)	961.9(12)	4143.5(7)	23.6(4)
C24	-2091(3)	2778.0(11)	3727.9(7)	16.9(3)
C25	-2258(3)	2554.3(12)	3110.6(7)	21.1(4)
C26	-737(3)	1811.4(12)	2941.8(7)	17.9(3)
C27	-1243(3)	906.5(13)	3052.4(7)	21.1(4)
C28	167(3)	208.4(12)	2929.6(8)	26.2(4)
C29	2117(3)	402.7(14)	2690.4(8)	27.7(4)
C30	2639(3)	1295.0(15)	2570.9(7)	27.5(4)
C31	1223(3)	1995.3(13)	2696.8(7)	23.1(4)

Table 3 Anisotropic Displacement Parameters (Å^2×10^3) for bcorr_a. The Anisotropic displacement factor exponent takes the form: $-2\pi^2[h^2a^{*2}U_{11}+2hka^*b^*U_{12}+\ldots]$.

**Atom**	**U_{11}**	**U_{22}**	**U_{33}**	**U_{23}**	**U_{13}**	**U_{12}**
N1	15.8(6)	10.9(6)	16.2(6)	-2.7(5)	0.6(5)	1.1(5)
C2	17.4(8)	11.6(7)	14.5(7)	-0.5(6)	1.5(6)	0.1(6)
C3	13.3(7)	12.9(7)	15.3(7)	-0.7(6)	1.7(6)	1.1(6)
C4	13.5(7)	13.3(7)	11.7(7)	-0.9(5)	-0.6(6)	0.5(6)
C5	15.1(7)	15.4(7)	15.5(7)	-1.2(6)	2.0(6)	0.1(6)
C6	14.5(7)	15.1(7)	18.3(7)	-4.3(6)	-0.3(6)	2.5(6)
C7	18.1(8)	12.1(7)	18.9(7)	-1.8(6)	-3.1(6)	3.9(6)
C8	18.6(8)	10.9(7)	16.6(7)	-0.5(6)	-1.2(6)	3.0(6)
C9	27.4(8)	16.7(8)	15.4(7)	1.3(6)	-0.9(7)	4.4(7)
C10	28.0(9)	18.1(8)	19.0(8)	5.6(6)	6.8(7)	2.9(7)
C11	16.8(8)	10.9(7)	25.5(8)	1.4(6)	2.7(7)	-0.2(6)
C12	19.4(8)	12.7(7)	17.3(7)	-0.7(6)	-0.6(6)	3.0(6)
C13	19.3(7)	12.1(7)	14.4(7)	1.0(6)	2.5(6)	1.4(6)
O14	22.6(6)	20.4(6)	36.9(7)	2.9(5)	6.1(6)	-5.2(5)
C15	21.1(9)	25.7(10)	46.1(12)	-7.9(8)	-1.0(8)	-5.2(7)

C16	16.9(7)	12.3(7)	21.5(8)	2.6(6)	2.8(7)	0.5(6)
N17	24.5(8)	18.7(7)	31.3(8)	1.2(6)	13.0(7)	2.0(6)
C18	14.6(7)	11.5(7)	12.6(7)	-0.8(5)	0.5(6)	0.1(6)
C19	18.4(7)	14.1(7)	12.8(7)	-0.4(6)	0.8(6)	0.5(6)
C20	17.5(7)	17.0(8)	12.0(7)	-0.2(6)	-1.5(6)	0.8(6)
O21	29.1(7)	19.1(6)	26.1(6)	4.9(5)	8.4(5)	0.9(5)
O22	23.3(6)	17.1(6)	20.7(6)	2.7(4)	6.3(5)	4.6(5)
C23	30.3(10)	19.7(8)	21.0(8)	0.9(7)	3.4(7)	10.8(7)
C24	14.1(7)	16.8(7)	19.8(8)	-3.5(6)	-0.1(6)	-0.9(6)
C25	22.0(8)	18.3(8)	23.0(8)	-6.5(6)	-9.3(7)	4.1(7)
C26	20.5(8)	19.5(8)	13.7(7)	-5.6(6)	-4.9(6)	1.8(7)
C27	21.3(8)	21.9(9)	20.1(8)	-3.3(7)	1.4(7)	1.0(7)
C28	36.3(10)	21.0(8)	21.3(8)	-3.0(7)	-0.3(8)	7.9(8)
C29	28.1(9)	35.8(10)	19.3(8)	-10.1(7)	-5.3(7)	13.0(8)
C30	18.5(8)	48.3(11)	15.7(8)	-10.9(8)	-0.5(6)	-1.0(8)
C31	27.3(9)	26.9(9)	15.0(7)	-6.2(7)	-3.0(7)	-6.8(7)

Table 4 Bond Lengths for bcorr_a.

**Atom**	**Atom**	**Length/Å**	**Atom**	**Atom**	**Length/Å**
N1	C2	1.464(2)	O14	C15	1.425(3)
N1	C6	1.471(2)	C16	N17	1.147(2)
N1	C7	1.4775(19)	C18	C19	1.541(2)
C2	C3	1.540(2)	C18	C24	1.540(2)
C3	C4	1.546(2)	C19	C20	1.505(2)
C3	C16	1.472(2)	C20	O21	1.205(2)
C4	C5	1.529(2)	C20	O22	1.345(2)
C4	C18	1.541(2)	O22	C23	1.441(2)
C5	C6	1.524(2)	C24	C25	1.538(2)
C7	C8	1.505(2)	C25	C26	1.508(2)
C8	C9	1.395(2)	C26	C27	1.395(3)
C8	C13	1.394(2)	C26	C31	1.392(3)
C9	C10	1.387(3)	C27	C28	1.388(3)
C10	C11	1.396(3)	C28	C29	1.384(3)
C11	C12	1.391(2)	C29	C30	1.384(3)
C11	O14	1.369(2)	C30	C31	1.395(3)
C12	C13	1.391(2)			

Table 5 Bond Angles for bcorr_a.

Atom	Atom	Atom	Angle/°	Atom	Atom	Atom	Angle/°
C2	N1	C6	110.21(12)	C12	C13	C8	121.98(15)
C2	N1	C7	110.13(13)	C11	O14	C15	117.03(14)
C6	N1	C7	109.63(13)	N17	C16	C3	177.57(19)
N1	C2	C3	110.12(13)	C19	C18	C4	112.99(12)
C2	C3	C4	111.48(13)	C24	C18	C4	111.08(13)
C16	C3	C2	108.50(13)	C24	C18	C19	111.09(13)
C16	C3	C4	110.16(13)	C20	C19	C18	110.68(13)
C5	C4	C3	107.48(12)	O21	C20	C19	125.94(15)
C5	C4	C18	114.91(13)	O21	C20	O22	123.17(15)
C18	C4	C3	112.76(13)	O22	C20	C19	110.89(13)
C6	C5	C4	111.21(13)	C20	O22	C23	116.80(13)
N1	C6	C5	110.10(13)	C25	C24	C18	112.67(14)
N1	C7	C8	113.15(13)	C26	C25	C24	112.14(14)
C9	C8	C7	121.95(15)	C27	C26	C25	119.71(16)
C13	C8	C7	120.36(15)	C31	C26	C25	122.27(17)
C13	C8	C9	117.68(15)	C31	C26	C27	117.97(16)
C10	C9	C8	121.52(16)	C28	C27	C26	121.33(18)
C9	C10	C11	119.58(15)	C29	C28	C27	120.02(18)
C12	C11	C10	120.15(15)	C28	C29	C30	119.54(17)
O14	C11	C10	115.91(16)	C29	C30	C31	120.31(18)
O14	C11	C12	123.94(16)	C26	C31	C30	120.82(18)
C13	C12	C11	119.08(15)				

Table 6 Hydrogen Atom Coordinates (Å×10^4) and Isotropic Displacement Parameters (Å^2×10^3) for bcorr_a.

Atom	*x*	*y*	*z*	U(eq)
H2A	2853.28	6177.35	3586.59	17
H2B	765.53	5931.28	3235.89	17
H3	2574.74	4789.21	4083.55	17
H4	-1066.41	4397.43	3400.7	15
H5A	-3139.3	4537.51	4207.03	18
H5B	-1049.18	4776.88	4557.38	18
H6A	-2676.27	5883.14	3677.39	19

H6B	-2894.38	6130.8	4317.98	19
H7A	-1212.7	7506.56	4177.13	20
H7B	-745.44	7332.95	3538.2	20
H9	2434.1	7963.51	3141.25	24
H10	5574.35	8796.57	3223.86	26
H12	5105.5	8652.52	4891.38	20
H13	2005.72	7789.92	4796.73	18
H15A	7284.82	9890.72	4810.51	46
H15B	9643.65	9878.76	4559.29	46
H15C	8634.51	8968	4807.61	46
H18	1094.73	3091.49	3615.82	15
H19A	1938.99	3482.11	4555	18
H19B	-461.77	3236.03	4724.84	18
H23A	3641.51	715.67	4517.63	35
H23B	4935.95	913.9	3962.51	35
H23C	2484.05	616.76	3933.33	35
H24A	-2115.35	2204.92	3941.27	20
H24B	-3347.29	3141.71	3838.83	20
H25A	-3735.01	2362.61	3025.43	25
H25B	-1948.66	3108.38	2893.79	25
H27	-2580.6	765.33	3215.07	25
H28	-207.15	-402.92	3009.68	31
H29	3090.74	-73.01	2608.75	33
H30	3968.13	1431.03	2402.39	33
H31	1599.86	2605.42	2614.37	28

Experimental

Single crystals of $C_{26}H_{32}N_2O_3$ [bcorr_a] were []. A suitable crystal was selected and [] on a SuperNova, Dual, Cu at zero, EosS2 diffractometer. The crystal was kept at 100.01(10) K during data collection. Using Olex2 [1], the structure was solved with the ShelXT [2] structure solution program using Intrinsic Phasing and refined with the ShelXL [3] refinement package using Least Squares minimisation.

1. Dolomanov, O.V., Bourhis, L.J., Gildea, R.J, Howard, J.A.K. & Puschmann, H. (2009), J. Appl. Cryst. 42, 339-341.
2. Sheldrick, G.M. (2015). Acta Cryst. A71, 3-8.
3. Sheldrick, G.M. (2015). Acta Cryst. C71, 3-8.

Crystal structure determination of [bcorr_a]

Crystal Data for $C_{26}H_{32}N_2O_3$ (M =420.53 g/mol): orthorhombic, space group $P2_12_12_1$ (no. 19), a = 6.27180(10) Å, b = 14.71860(10) Å, c = 24.2813(2) Å, V = 2241.46(4) Å^3, Z = 4, T = 100.01(10) K, μ(CuKα) = 0.645 mm^{-1}, $Dcalc$ = 1.246 g/cm^3, 43306 reflections measured (7.022° ≤ 2Θ ≤ 145.98°), 4462 unique (R_{int} = 0.0424, R_{sigma} = 0.0200) which were used in all calculations. The final R_1 was 0.0288 (I > 2σ(I)) and wR_2 was 0.0743 (all data).

Refinement model description

Number of restraints - 0, number of constraints - unknown.

Details:

1. Fixed Uiso
 At 1.2 times of:
  All C(H) groups, All C(H,H) groups
 At 1.5 times of:
  All C(H,H,H) groups
2.a Ternary CH refined with riding coordinates:
 C3(H3), C4(H4), C18(H18)
2.b Secondary CH2 refined with riding coordinates:
 C2(H2A,H2B), C5(H5A,H5B), C6(H6A,H6B), C7(H7A,H7B), C19(H19A,H19B), C24(H24A, H24B), C25(H25A,H25B)
2.c Aromatic/amide H refined with riding coordinates:
 C9(H9), C10(H10), C12(H12), C13(H13), C27(H27), C28(H28), C29(H29), C30(H30), C31(H31)
2.d Idealised Me refined as rotating group:
 C15(H15A,H15B,H15C), C23(H23A,H23B,H23C)
